# The RalGAPα1–RalA signal module protects cardiac function through regulating calcium homeostasis

**DOI:** 10.1038/s41467-022-31992-z

**Published:** 2022-07-25

**Authors:** Sangsang Zhu, Chao Quan, Ruizhen Wang, Derong Liang, Shu Su, Ping Rong, Kun Zhou, Xinyu Yang, Qiaoli Chen, Min Li, Qian Du, Jingzi Zhang, Lei Fang, Hong-Yu Wang, Shuai Chen

**Affiliations:** 1 State Key Laboratory of Pharmaceutical Biotechnology and MOE Key Laboratory of Model Animal for Disease Study, Department of Cardiology, Nanjing Drum Tower Hospital, The Affiliated Hospital of Nanjing University Medical School, Model Animal Research Center, Nanjing University, Nanjing, China; 2 School of Medicine, Nanjing University, Nanjing, China

**Keywords:** Heart failure, Calcium signalling, Preclinical research

## Abstract

Sarcoplasmic/endoplasmic reticulum calcium ATPase SERCA2 mediates calcium re-uptake from the cytosol into sarcoplasmic reticulum, and its dysfunction is a hallmark of heart failure. Multiple factors have been identified to modulate SERCA2 activity, however, its regulation is still not fully understood. Here we identify a Ral-GTPase activating protein RalGAPα1 as a critical regulator of SERCA2 in cardiomyocytes through its downstream target RalA. RalGAPα1 is induced by pressure overload, and its deficiency causes cardiac dysfunction and exacerbates pressure overload-induced heart failure. Mechanistically, RalGAPα1 regulates SERCA2 through direct interaction and its target RalA. Deletion of RalGAPα1 decreases SERCA2 activity and prolongs calcium re-uptake into sarcoplasmic reticulum. GDP-bound RalA, but not GTP-bound RalA, binds to SERCA2 and activates the pump for sarcoplasmic reticulum calcium re-uptake. Overexpression of a GDP-bound RalA^S28N^ mutant in the heart preserves cardiac function in a mouse model of heart failure. Our findings have therapeutic implications for treatment of heart failure.

## Abstract (web summary)

Here the authors show that a RalGAPα1-RalA signal nexus regulates calcium homeostasis in cardiomyocytes via the calcium pump SERCA2a, which plays a protective role to maintain cardiac function under pressure overload conditions.

## Introduction

Hypertension and its associated cardiovascular and cardiac diseases are worldwide leading causes of death^1^. Pressure overload due to hypertension causes cardiac dysfunction and leads to the development of cardiomyopathy and heart failure. Pressure overload elicits structural and molecular alterations in the heart, whose roles in the pathogenesis of hypertension-induced cardiomyopathy are not clearly understood^2^. A better understanding of such pathological mechanisms may help to develop drugs to treat hypertension-induced cardiomyopathy.

Calcium (Ca^2+^) cycling between the sarcoplasmic reticulum (SR) and cytosol in cardiomyocytes dictates cardiac contractile activities^3^. Pressure overload impairs Ca^2+^ cycling in the heart and causes an increase of cytosolic Ca^2+^, which in turn attenuates cardiac contractility^4^. Sarcoplasmic/endoplasmic reticulum Ca^2+^ ATPase 2 (SERCA2) is a critical enzyme mediating SR re-uptake of Ca^2+^ from the cytosol, whose dysfunction is evident in failing hearts^5^. SERCA2 function is under negative and positive control by multiple mechanisms including phospholamban-binding^6^, phosphorylation^7,8^ and SUMOylation^9^. Binding of phospholamban imposes an inhibitory effect on SERCA2 and slows down re-uptake of Ca^2+^ into the SR^6^. In contrast, Thr^484^ phosphorylation of SERCA2 by striated muscle preferentially expressed protein kinase (SPEG) enhances its Ca^2+^ transport activity^7,8^. Similarly, SUMOylation of SERCA2 preserves its ATPase activity and stability, which is decreased in failing heart^9^. Given the importance of SERCA2 in regulation of Ca^2+^ homeostasis, restoration of SERCA2 function through its expression or regulation has been proposed as a potential strategy to treat heart failure^6^.

Ral-GTPases consisting of RalA and RalB are key regulators of diverse cellular processes including exocytosis^10^, proliferation^11^ and autophagy^12^. Although they exhibit certain redundancy, the two Ral-GTPases can have distinct functions in many processes^13^. The intrinsic GTPase activities of RalA and RalB are enhanced by their upstream regulator, Ral-GTPase activating protein (RalGAP) complexes, which converts these two small G proteins from a GTP-bound state to a GDP-bound form^14,15^. Two catalytic RalGAPα1 and α2 subunits bind to a common regulatory RalGAPβ subunit to form RalGAP complex-1 and 2, respectively^14^. The two RalGAP complexes exhibit distinct tissue distribution with complex-1 as the dominant one in skeletal muscle and complex-2 as the major one in adipose tissue^16^. Both RalGAP complexes regulate trafficking of glucose transporter GLUT4 in skeletal muscle and adipose tissues^17,18^. Besides, the RalGAP complex-1 mediates insulin-induced trafficking of fatty acid translocase CD36 in the skeletal muscle^17^. Both RalGAP complexes are expressed in the heart, however their functions in this tissue remain unknown so far.

In this study, we identify RalGAPα1 as a critical regulator of SERCA2 in cardiomyocytes through the GDP-bound RalA. We generate a cardiomyocyte-specific RalGAPα1 knockout mouse model and demonstrate that RalGAPα1 plays a protective role in pressure overloaded hearts through regulating SERCA2.

## Results

### Pressure overload increases protein expression of RalGAPα1 complex in the heart

**Fig. 1:**
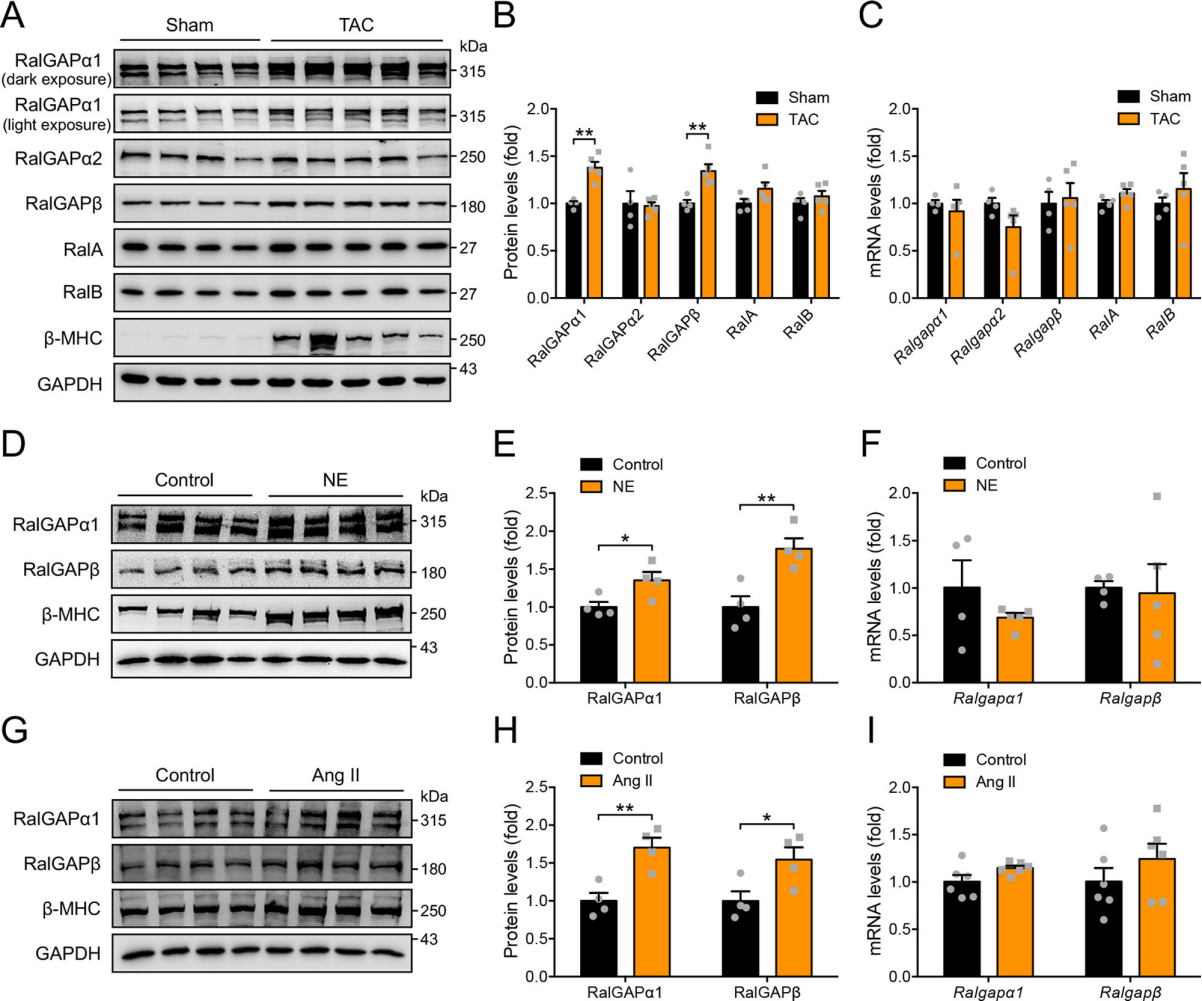
Expression of RalGAP−Ral signaling components in the heart and cardiomyocytes. **A**, **B** Protein expression of RalGAP complexes and their downstream Ral small G proteins in the hearts of 2-month-old WT male mice that were subjected to TAC or sham operation for 12 days. GAPDH was used as a loading control. B quantitation of blots shown in **A**. *n* = 4 (Sham) and 5 (TAC). *p* = 0.0011 (RalGAPα1), 0.843 (RalGAPα2), 0.0055 (RalGAPβ), 0.113 (RalA) and 0.386 (RalB). **C** mRNA expression of RalGAP complexes and their downstream Ral small G proteins in the heart of 2-month-old WT male mice that were subjected to TAC or sham operation for 12 days. *n* = 4 (Sham) and 5 (TAC). *p* = 0.578 (*Ralgapα1*), 0.138 (*Ralgapα2*), 0.792 (*Ralgapβ*), 0.112 (*RalA*) and 0.457 (*RalB*). **D**, **E** Protein expression of RalGAPα1/β complexes and β-MHC in NRVCs stimulated with or without norepinephrine (NE). **E** quantitation of blots shown in **D**. *n* = 4. *p* = 0.035 (RalGAPα1) and 0.0078 (RalGAPβ). **F** mRNA expression of RalGAP complexes in NRVCs stimulated with or without NE. *n* = 4 (Control) and 5 (NE). *p* = 0.262 (*Ralgapα1*) and 0.877 (*Ralgapβ*). **G**, **H** Protein expression of RalGAPα1/β complexes and β-MHC in NRVCs stimulated with or without Angiotensin II (Ang II). H, quantitation of blots shown in G. *n* = 4. *p* =0.0059 (RalGAPα1) and 0.039 (RalGAPβ). **I** mRNA expression of RalGAP complexes in NRVCs stimulated with or without Ang II. *n* = 6. *p* = 0.085 (*Ralgapα1*) and 0.290 (*Ralgapβ*). The data are given as the mean ± SEM. Statistical analyses were carried out using two-sided t-test. One-asterisk indicates *p* < 0.05, and two-asterisk indicates *p* < 0.01. Source data are provided as a Source Data file.

To find out whether the two RalGAP complexes and their downstream Ral small G proteins might have roles in hypertension-induced cardiomyopathy, we first examined their expression in pressure-overloaded hearts that were subjected to transverse aortic constriction (TAC). As expected, pressure-overload increased βMHC in the heart at the protein level (Fig. 1A). The mRNA levels of *Ralgapα1* and *Ralgapβ* were comparable in the sham-operated and TAC-treated heart whereas their protein levels were significantly increased in the heart subjected to TAC (Fig. 1A–C). In contrast, neither mRNA nor protein levels of RalGAPα2 changed in the TAC-treated heart (Fig. 1A–C). The downstream targets of RalGAP complexes, namely RalA and RalB, remained normal in the TAC-treated heart at both mRNA and protein levels (Fig. 1A–C). Angiotensin-II (Ang-II) and norepinephrine (NE) are two key regulators for the neurohumoral responses to pressure overload-induced cardiomyopathy^19^. Treatment with NE increased RalGAPα1 protein but not its mRNA in primary neonatal rat ventricular cardiomyocytes (NRVCs) (Fig. 1D–F). Interestingly, turnover of RalGAPα1 protein was slower in NE-treated NRVCs than in control cells after the addition of cycloheximide (Supplementary Fig. 1A, B). These results suggest that NE elevated RalGAPα1 protein in cardiomyocytes most likely through increasing its stability. Similarly, stimulation with Ang-II did not change *Ralgapα1* mRNA but increased its protein level in primary NRVCs (Fig. 1G–I). Together, these data suggest that the RalGAPα1 complex might play a role in pressure overload-induced cardiomyopathy.

### RalGAPα1 deficiency causes cardiac dysfunction and exacerbates TAC-induced cardiomyopathy

**Fig. 2:**
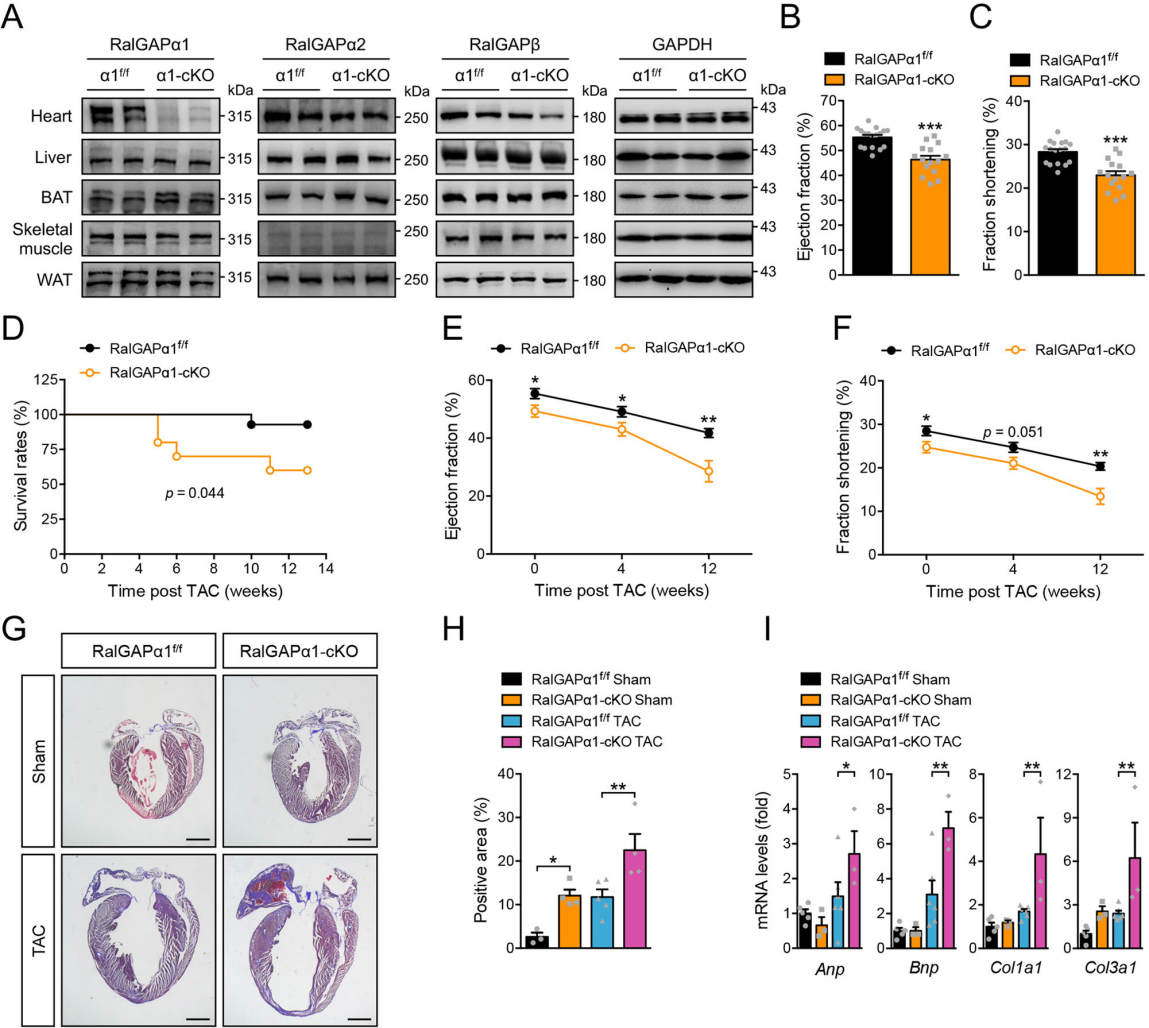
Cardiac function and remodeling in the RalGAPα1-cKO heart under basal and TAC conditions. **A** Protein expression of RalGAPα1, RalGAPα2, and RalGAPβ in various tissues of male RalGAPα1-cKO mice (3-month-old). GAPDH was used as a loading control. B-C. Ejection fraction **B** and fractional shortening **C** were measured via echocardiography in the male RalGAPα1-cKO and RalGAPα1^f/f^ mice (3-month-old). *n* = 15 (RalGAPα1-cKO) and 17 (RalGAPα1^f/f^). *p* = 1.99e-5 (EF) and 2.73e-5 (FS). **D** Survival rates of the male RalGAPα1-cKO and RalGAPα1^f/f^ mice after TAC. *n* = 10 (RalGAPα1-cKO) and 14 (RalGAPα1^f/f^). *p* = 0.044. **E**, **F** Ejection fraction **E** and fractional shortening **F** in the male RalGAPα1-cKO and RalGAPα1^f/f^ mice before and after TAC surgery. TAC surgery was performed in 3-month-old mice. *n* = 11 (RalGAPα1-cKO) and 14 (RalGAPα1^f/f^). EF: *p* = 0.033 (0 w), 0.049 (4 w) and 0.0010 (12 w). FS: *p* = 0.033 (0 w), 0.051 (4 w) and 0.0011 (12 w). **G**, **H** Masson’s staining of the heart sections in the sham- or TAC-operated RalGAPα1-cKO and RalGAPα1^f/f^ male mice (6-month-old). **G** representative images. **H** quantitation of positive staining area. *n* = 3 (RalGAPα1^f/f^ Sham), 4 (RalGAPα1-cKO Sham), 5 (RalGAPα1^f/f^ TAC) and 4 (RalGAPα1-cKO TAC). Bars indicate 1 mm in length. *p* = 0.020 (RalGAPα1^f/f^ Sham vs RalGAPα1-cKO Sham) and 0.0046 (RalGAPα1^f/f^ TAC vs RalGAPα1-cKO TAC). **I** Expression of *Anp*, *Bnp*, *Col1a1* and *Col3a1* mRNA in the hearts of sham- or TAC-operated RalGAPα1-cKO and RalGAPα1^f/f^ male mice (6-month-old). *n* = 5 (RalGAPα1^f/f^ Sham), 6 (RalGAPα1^f/f^ TAC), 3 (RalGAPα1-cKO Sham), and 3 (RalGAPα1-cKO TAC). *p* = 0.048 (*Anp*), 0.0021 (*Bnp*), 0.0075 (*Col1a1*) and 0.0079 (*Col3a1*) (RalGAPα1^f/f^ TAC vs RalGAPα1-cKO TAC). The data are given as the mean ± SEM. Statistical analyses were carried out using two-sided t-test for B, C, E and F, and two-way ANOVA for H and I. One-asterisk indicates *p* < 0.05, two-asterisk indicates *p* < 0.01 and three-asterisk indicates *p* < 0.001. Source data are provided as a Source Data file.

Cardiomyocytes and fibroblasts are two cell populations in the heart. We wondered whether the two RalGAP complexes might be differentially expressed in these two cell types although they both are found in the heart^16^. Both RalGAPα1 and α2 were preferentially expressed in cardiomyocytes rather than in fibroblasts from hearts (Supplementary Fig. 1C). To elucidate the role of RalGAPα1 in regulation of cardiac function, we generated a RalGAPα1 cardiomyocyte-specific deletion mouse model (RalGAPα1-cKO) through mating RalGAPα1^f/f^ with an αMHC-Cre mouse^20^. As expected, RalGAPα1 was diminished in the heart but unaltered in the other tissues including skeletal muscle, liver, brown adipose tissue and white adipose tissue (Fig. 2A). Cardiomyocyte-specific deletion of RalGAPα1 did not affect the expression of RalGAPα2 (Fig. 2A). Since the stability of RalGAPβ depends on RalGAPα, RalGAPβ exhibited a moderate decrease in the heart but remained normal in the other tissues analyzed (Fig. 2A). The mRNA levels of RalGEFs in the heart were comparable between the two genotypes (Supplementary Fig. 2A). These data demonstrate that the RalGAPα1-cKO mice and their derived cardiomyocytes are suitable for investigation of possible functions of RalGAPα1 in the heart. Whole-body glucose homeostasis was not impaired in the RalGAPα1-cKO mice as evidenced by normal tolerance to glucose challenge (Supplementary Fig. 3A). Cardiac energy metabolism was also normal in the RalGAPα1-cKO mice with unaltered expression of mitochondrial respiratory chain (Supplementary Fig. 3B–E). Moreover, phosphorylation of PKB and MAPKs was not affected in the RalGAPα1-cKO heart (Supplementary Fig. 2B). Interestingly, we found that deletion of RalGAPα1 in the heart impaired cardiac function in mice. Both ejection fraction (EF) and fractional shortening (FS) were significantly lower in the RalGAPα1-cKO mice than in the RalGAPα1^f/f^ littermates (Fig. 2B, C). We then subjected the RalGAPα1-cKO and RalGAPα1^f/f^ mice to TAC surgery. Importantly, the RalGAPα1-cKO mice exhibited a higher death rate than the RalGAPα1^f/f^ littermates after the surgery (Fig. 2D). Again, both EF and FS were lower in the remaining RalGAPα1-cKO mice than in the control mice (Fig. 2E, F). Deletion of RalGAPα1 also exacerbated TAC-induced cardiac remodeling with upregulation of cardiac fibrosis and increased expression of heart failure markers such as *Anp*, *Bnp*, *Col1a1* and *Col3a1* in the RalGAPα1-cKO hearts as compared to the control hearts (Fig. 2G–I). These data demonstrate that upregulation of the RalGAPα1 complex plays a protective role in the pressure-overloaded heart.

### Identification of SERCA2 as a target interacting with the RalGAPα1 complex

**Fig. 3:**
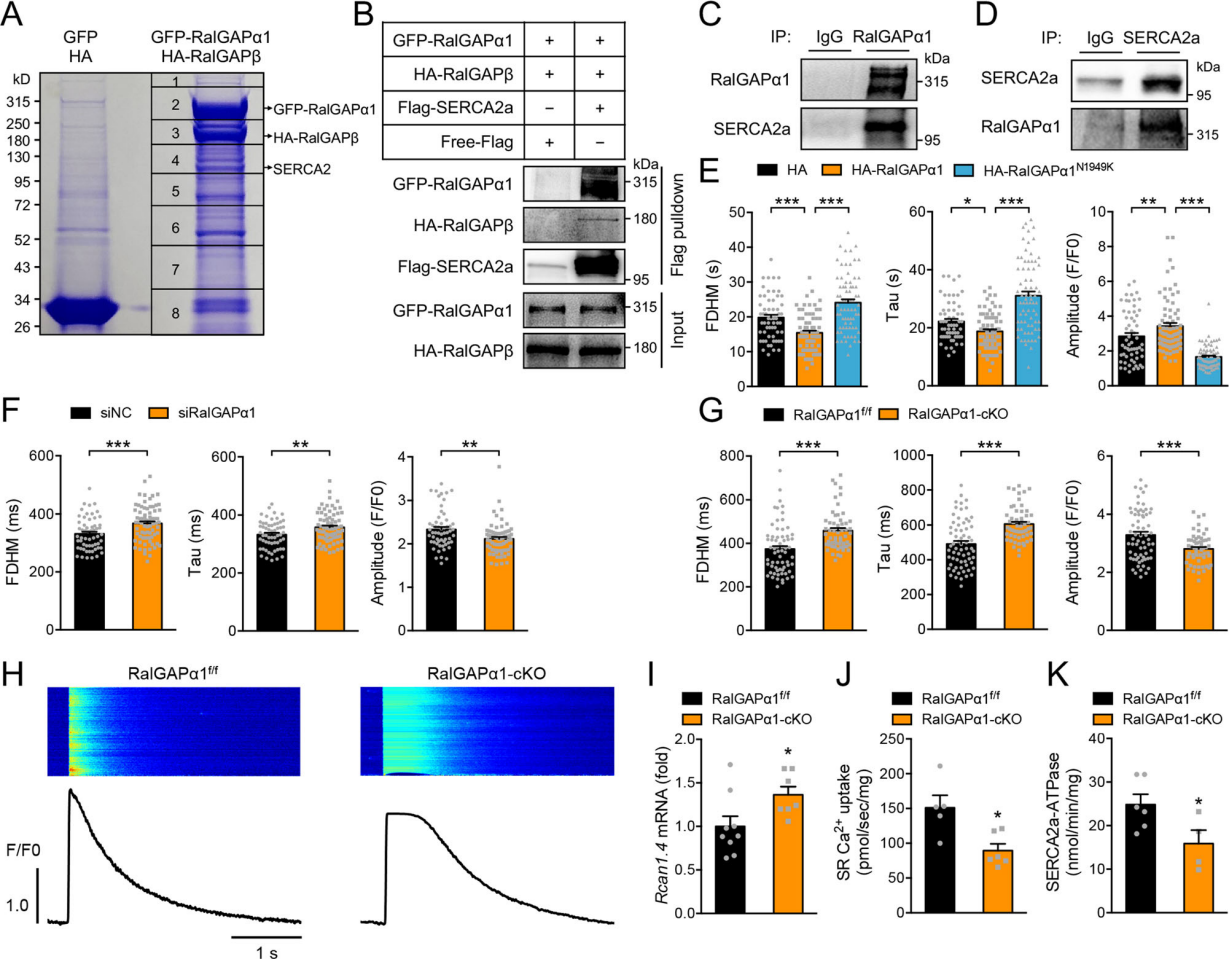
Identification of RalGAPα1 as a regulator of SERCA2a. **A** Identification of proteins associated with RalGAPα1. GFP-RalGAPα1 was expressed in HEK293 cells together with HA-RalGAPβ, and empty vectors were expressed in cells as a control. Immunoprecipitation was carried out using the GFP-binder, and proteins in the immunoprecipitates were separated via SDS-PAGE and identified via mass-spectrometry. **B** GFP-RalGAPα1/HA-RalGAPβ complex was co-expressed with Flag-SERCA2a or free Flag in HEK293 cells. After immunoprecipitation with the Flag antibody, GFP-RalGAPα1/HA-RalGAPβ complex was detected in the immunoprecipitates via western blot. **C** Endogenous RalGAPα1 was immunoprecipitated from heart lysates, and endogenous SERCA2a was detected in the immunoprecipitates via western blot. **D** Endogenous SERCA2a was immunoprecipitated from heart lysates, and endogenous RalGAPα1 was detected in the immunoprecipitates via western blot. **E** Ca^2+^ transients in HEK293 cells expressing mCherry-SERCA2a together with HA- RalGAPα1 or HA-RalGAPα1^N1949K^ proteins. Ca^2+^ transients were recorded using confocal microscopy in cells that were stimulated with ATP. *n* = 59 (HA-vector), 75 (HA-RalGAPα1), and 73 (HA-RalGAPα1^N1949K^). FDHM: *p* = 0.0003 (HA-vector vs HA-RalGAPα1), and *p* < 0.0001 (HA-RalGAPα1 vs HA-RalGAPα1^N1949K^). Tau: *p* = 0.031 (HA-vector vs HA-RalGAPα1), and *p* < 0.0001 (HA-RalGAPα1 vs HA-RalGAPα1^N1949K^). Amplitude: *p* = 0.0094 (HA-vector vs HA-RalGAPα1), and *p* < 0.0001 (HA-RalGAPα1 vs HA-RalGAPα1^N1949K^). **F** Ca^2+^ transients in RalGAPα1-depleted neonatal rat cardiomyocytes upon field stimulation. Amplitude, FDHM and Tau of Ca^2+^ transients were quantified from 58 (siNC) and 72 (siRalGAPα1) cells. *p* = 0.0008 (FDHM), 0.0054 (Tau) and 0.0023 (Amplitude). **G**, **H** Ca^2+^ transients in primary cardiomyocytes isolated from the male RalGAPα1^f/f^ and RalGAPα1-cKO mice (2-month-old) upon field stimulation. Amplitude, FDHM and Tau of Ca^2+^ transients were measured in 66 RalGAPα1^f/f^ cells (3 mice) and 56 RalGAPα1-cKO cells (2 mice), respectively. **H** representative Ca^2+^ transient images and curves. *p* = 1.46e-5 (FDHM), 7.84e-7 (Tau) and 0.00041 (Amplitude). **I** Expression of *Rcan1.4* mRNA in the hearts of male RalGAPα1-cKO and RalGAPα1^f/f^ mice (3-month-old). *n* = 9 (RalGAPα1^f/f^) and 7 (RalGAPα1-cKO). *p* = 0.036. **J**, **K** SERCA2a Ca^2+^-transporting activity **J** and ATPase activity **K** in microsomes isolated from hearts of male RalGAPα1-cKO and RalGAPα1^f/f^ mice (2-month-old). SR Ca^2+^-uptake: *n* = 5 (RalGAPα1^f/f^) and 6 (RalGAPα1-cKO). SERCA2a-ATPase: *n* = 6 (RalGAPα1^f/f^) and 4 (RalGAPα1-cKO). *p* = 0.011 (SR Ca^2+^-uptake) and 0.048 (SERCA2a-ATPase). The data are given as the mean ± SEM. Statistical analyses were carried out using one-way ANOVA for 3E and two-sided t-test for 3**F**–**G**, **I**–**K** One-asterisk indicates *p* < 0.05, two-asterisk indicates *p* < 0.01 and three-asterisk indicates *p* < 0.001. Source data are provided as a Source Data file.

To gain mechanistic insights how the RalGAPα1 complex regulates cardiac function, we took a proteomics approach to identify its interacting proteins. To this end, we expressed GFP-RalGAPα1 together with HA-RalGAPβ in HEK293 cells, and subsequently immunoprecipitated it using GFP-Trap beads. A number of proteins were co-immunoprecipitated with GFP-RalGAPα1, which were identified via mass-spectrometry (Fig. 3A, Supplementary Data 1). As expected, RalGAPβ was found in the immunoprecipitates as a known binding partner of RalGAPα1 (Fig. 3A). Interestingly, SERCA2, the key regulator of cardiac Ca^2+^ homeostasis, was identified as a potential interacting partner of RalGAPα1 (Fig. 3A, Supplementary Data 1). We further verified the presence of SERCA2 in the GFP-RalGAPα1 immunoprecipitates via Western blot (Fig. 3B). Moreover, the endogenous SERCA2 was co-immunoprecipitated with the endogenous RalGAPα1 that was immunoprecipitated from heart lysates using a specific antibody (Fig. 3C). In a reciprocal co-immunoprecipitation exeriment, endogenous RalGAPα1 was also found in the immunoprecipitates of endogenous SERCA2 from heart lysates (Fig. 3D).

### The RalGAPα1 complex regulates SR Ca^2+^ re-uptake in cardiomyocytes

Interaction of the RalGAPα1 complex with SERCA2 suggests that it might regulate Ca^2+^ homeostasis through control of SR Ca^2+^ re-uptake. To examine this possibility, we overexpressed RalGAPα1 in HEK293 cells and measured Ca^2+^ transients that were elicited by addition of ATP. The full duration at half maximum (FDHM) and time constant Tau of Ca^2+^ transients are two measures reflecting the rates of Ca^2+^ re-uptake into the ER, and the peak is a measure of cytosolic Ca^2+^. Overexpression of RalGAPα1 caused a significant decrease of the FDHM and Tau of Ca^2+^ transients in HEK293 cells, suggesting an acceleration of Ca^2+^ re-uptake into the ER (Fig. 3E). The peak of Ca^2+^ transients was significantly increased in cells overexpressing RalGAPα1, indicating a decrease in cytosolic Ca^2+^ (Fig. 3E). We then knocked-down RalGAPα1 in NRVCs using small interfering RNA (siRNA), and examined the effects of RalGAPα1 depletion on Ca^2+^ transients. The FDHM and Tau of Ca^2+^ transients were significantly increased while their peaks were depressed in RalGAPα1-depleted NRVCs (Fig. 3F).

Cardiomyocyte-specific deletion of RalGAPα1 did not affect mRNA levels of *Ltcc*, *Ncx* and *Ryr2* in the heart (Supplementary Fig. 2C). Expression of SERCA2a was normal in the heart of RalGAPα1-cKO mice at both mRNA and protein levels (Supplementary Fig. 2C, D). We then isolated primary cardiomyocytes and analyzed Ca^2+^ homeostasis in these cells. Transverse tubules (t-tubules) are invaginated sarcolemma in cardiomyocytes, which contain membrane microdomains enriched with ion channels and transporters^21^. T-tubules form a branched and interconnected network, and their regularity was comparable between the RalGAPα1-deficient and control cells (Supplementary Fig. 4A, B). We measured Ca^2+^ transients in primary cardiomyocytes, which were elicited by electrical stimulation. Interestingly, the FDHM and Tau of Ca^2+^ transients were significantly increased in RalGAPα1-deficient cardiomyocytes (Fig. 3G, H). This prolonged Ca^2+^ re-uptake into the SR resulted in elevation of cytosolic Ca^2+^ as evidenced by a diminution of the amplitude of Ca^2+^ transients (Fig. 3G, H). Cytosolic Ca^2+^ is an inducer of Rcan1.4 expression through the Calcineurin-NFAT pathway^22^. In agreement with elevation of cytosolic Ca^2+^, Rcan1.4 expression was significantly increased in the RalGAPα1-cKO heart as compared to the RalGAPα1^f/f^ control heart (Fig. 3I). The frequency of Ca^2+^ sparks was largely unchanged in the RalGAPα1-deficient cardiomyocytes as compared to that in the control cells (Supplementary Fig. 4C), suggesting that RalGAPα1 deletion might not affect spontaneous Ca^2+^ release from the SR. In agreement with the prolonged Ca^2+^ re-uptake in the Ca^2+^ transient assay, both SERCA2-ATPase activity and SR Ca^2+^ transport were significantly decreased in microsomes isolated from the RalGAPα1-deficient heart as compared to the controls (Fig. 3J, K).

Together, these data demonstrate that RalGAPα1 controls Ca^2+^ homeostasis in cardiomyocytes through regulating SERCA2-mediated SR Ca^2+^ re-uptake. RalGAPα1 deficiency prolongs SR Ca^2+^ re-uptake in cardiomycotytes, which might underlie cardiac dysfunction in the RalGAPα1-cKO mice.

### GDP-bound RalA regulates SERCA2 at the downstream of RalGAPα1

**Fig. 4:**
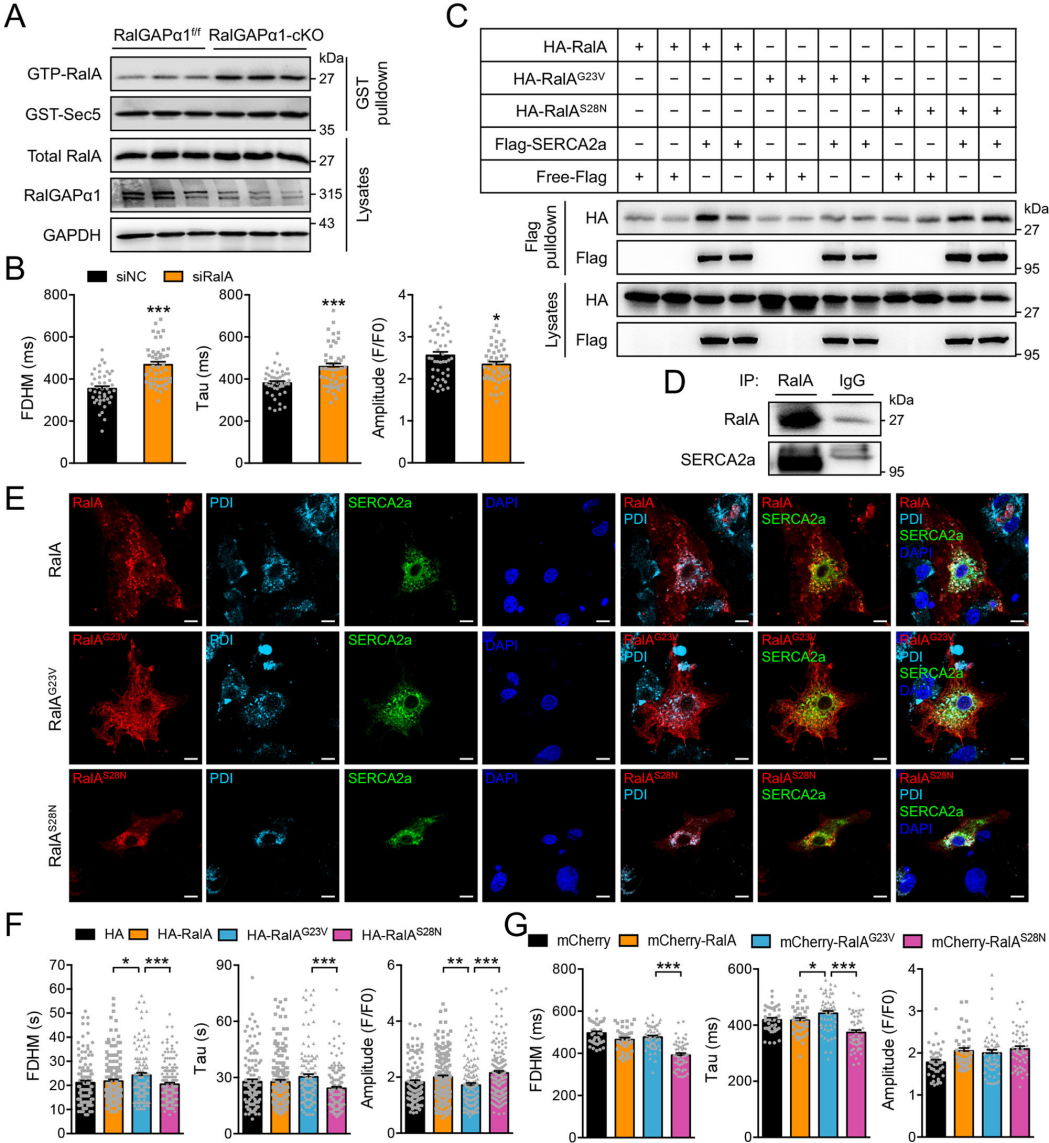
Effects of RalA on Ca2+ homeostasis. **A** GTP-bound RalA was measured in lysates of RalGAPα1^f/f^ and RalGAPα1-cKO hearts (2-month-old, female). RalA-GTP was pulled-down from heart lysates using GST-Sec5N as a bait, and detected via western blot using the anti-RalA antibody. Total RalA and RalGAPα1 were detected in heart lysates using GAPDH as a loading control. **B** Ca^2+^ transients in RalA-knockdown neonatal rat cardiomyocytes upon field stimulation. Amplitude, FDHM and Tau of Ca^2+^ transients were quantified from 43 (siNC) and 45 (siRalA) cells. *p* = 3.40e-8 (FDHM), 4.23e-5 (Tau) and 0.044 (Amplitude). **C** Interaction of RalA with SERCA2a was examined via co-immunoprecipitation. HA-RalA, HA-RalA^G23V^ and HA-RalA^S28N^ were co-expressed with Flag-SERCA2a or free Flag in HEK293 cells, respectively. Co-immunoprecipitated was detected via western blot. After immunoprecipitation with the Flag antibody, HA-RalA WT or mutant proteins were detected in the immunoprecipitates via western blot. **D** Endogenous RalA was immunoprecipitated from heart lysates, and endogenous SERCA2a was detected in the immunoprecipitates via western blot. **E** Colocalization of RalA and SERCA2a in cardiomyocytes. mCherry-RalA, mCherry-RalA^G23V^ and mCherry-RalA^S28N^ were co-expressed with GFP-SERCA2a in neonatal rat cardiomyocytes. Endogenous PDI was stained with the specific antibody and used as a SR marker. Bars indicate 10 μm in length. **F** Ca^2+^ transients were measured in HEK293 cells expressing mCherry-SERCA2a together with HA vector, HA-RalA, HA-RalA^G23V^, or HA-RalA^S28N^ proteins after stimulation with ATP. Amplitudes, FDHM and Tau of Ca^2+^ transients were quantified from 120 (HA vector), 177 (HA-RalA), 117 (HA-RalA^G23V^), and 170 (HA-RalA^S28N^) cells. FDHM: *p* = 0.029 (HA-RalA vs HA-RalA^G23V^) and 0.0010 (HA-RalA^G23V^ vs HA-RalA^S28N^). Tau: *p* = 0.093 (HA-RalA vs HA-RalA^G23V^) and 0.0001 (HA-RalA^G23V^ vs HA-RalA^S28N^). Amplitude: *p* = 0.0050 (HA-RalA vs HA-RalA^G23V^), and *p* < 0.0001 (HA-RalA^G23V^ vs HA-RalA^S28N^). G. Ca^2+^ transients in neonatal rat cardiomyocytes expressing mCherry-RalA, mCherry-RalA^G23V^, mCherry-RalA^S28N^ or mCherry vector upon field stimulation. Amplitudes, FDHM and Tau of Ca^2+^ transients were quantified from 40 (vector), 38 (mCherry-RalA), 54 (mCherry-RalA^G23V^), and 51 (mCherry-RalA^S28N^) cells. FDHM: *p* = 0.333 (mCherry-RalA vs mCherry-RalA^G23V^), and *p* < 0.0001 (mCherry-RalA^G23V^ vs mCherry-RalA^S28N^). Tau: *p* = 0.039 (mCherry-RalA vs mCherry-RalA^G23V^), and *p* < 0.0001 (mCherry-RalA^G23V^ vs mCherry-RalA^S28N^). Amplitude: *p* = 0.611 (mCherry-RalA vs mCherry-RalA^G23V^) and 0.292 (mCherry-RalA^G23V^ vs mCherry-RalA^S28N^). The data are given as the mean ± SEM. Statistical analyses were carried out using two-sided t-test for **B**, and one-way ANOVA for **F**, **G**. One-asterisk indicates *p* < 0.05, and three-asterisk indicates *p* < 0.001. Source data are provided as a Source Data file.

We next examined the possible mechanism how RalGAPα1 regulates SERCA2. RalGAPα1 possesses a functional GAP domain with the Asn^1949^ as a key residue for its activity^16^. When an Asn^1949^Lys mutation was introduced to inactivate its GAP activity, RalGAPα1 lost its ability to accelerate Ca^2+^ re-uptake into the ER in the Ca^2+^ transient assay. The RalGAPα1^Asn1949Lys^ mutant was neither able to decrease the FDHM and Tau nor to increase the amplitude of Ca^2+^ transients as compared to the wild-type RalGAPα1 (Fig. 3E). As a GAP for RalA and RalB, RalGAPα1 can convert these two small G proteins from their GTP-bound form to GDP-bound form in vitro^14^. Expression of RalA and RalB in the heart was comparable between the RalGAPα1-cKO and RalGAPα1^f/f^ mice, whereas the GTP-bound form of RalA, but not the GTP-bound RalB, was increased in the RalGAPα1-deficient heart (Fig. 4A, Supplementary Fig. 5A). Cardiac expression of a GTP-bound RalA^G23V^ mutant using a recombinant adeno-associated virus (rAAV)-mediated gene delivery system did not cause impairment of cardiac function in mice, suggesting that the increase in GTP-bound RalA most likely did not account for cardiac dysfunction in the RalGAPα1-cKO mice (Supplementary Fig. 6A–D). Presumably, the GDP-bound form of RalA was decreased in the RalGAPα1-deficient heart. Notably, knockdown of RalA, but not RalB, increased the FDHM and Tau, and depressed the amplitude of Ca^2+^ transients in primary neonatal rat cardiomyocytes (Fig. 4B, Supplementary Fig. 5B). In agreement, we found that RalA, but not RalB, interacted with SERCA2 when co-expressed in HEK293 cells (Fig. 4C, Supplementary Fig. 5C). At the endogenous protein level, SERCA2a was detected in immunoprecipitates of RalA from heart lysates (Fig. 4D). Interestingly, a GDP-bound RalA^S28N^ mutant but not the GTP-bound RalA^G23V^ mutant interacted with SERCA2 when co-expressed in cells (Fig. 4C). In contrast, both the GDP-bound RalA^S28N^ and GTP-bound RalA^G23V^ mutants interacted with RalGAPα1 in a co-immunoprecipitation assay (Supplementary Fig. 7A–C, Supplementary Fig. 8A, B) and in an in vitro pulldown assay (Supplementary Fig. 8C, D). Moreover, both GTPγS-loaded and GDP-loaded RalA interacted with purified RalGAPα1 in the in vitro pulldown assay (Supplementary Fig. 8E, F). Interaction region mapping revealed that both RalA^S28N^ and RalA^G23V^ mutants bound to a region on RalGAPα1 spanning from Val^1133^ to Pro^1754^ that precedes its GAP domain (Supplementary Figs. 7A–C and 8A). Fragmentation experiments showed that multiple regions on SERCA2a might be involved in interaction with RalA and RalGAPα1 (Supplementary Fig. 9A–C). RalGAPα1 and RalA patially colocalised with SERCA2a in primary neonatal rat cardiomyocytes (Supplementary Fig. 9D). The GTP-bound RalA^G23V^ exhibited a filamentous structure when expressed in primary neonatal rat cardiomyocytes (Fig. 4E), which is consistent with its role in regulating vesicle trafficking through interacting with myosin^23^. The GDP-bound RalA^S28N^ was mainly associated with SR and displayed a high degree of colocalization with SERCA2a (Fig. 4E). More importantly, expression of the RalA^S28N^ mutant, but not the RalA^G23V^ mutant, decreased the FDHM and Tau, and increased the amplitude of Ca^2+^ transients in HEK293 cells (Fig. 4F) and in primary neonatal rat cardiomyocytes (Fig. 4G), suggesting that the GDP-bound form of RalA accelerates Ca^2+^ re-uptake into the SR. In contrast, neither a GTP-bound RalB^Q72L^ mutant nor a GDP-bound RalB^S28N^ mutant affected Ca^2+^ transients when expressed in primary neonatal rat cardiomyocytes (Supplementary Fig. 5D).

Together, these data show that RalGAPα1 regulates SERCA2a through the GDP-bound form of RalA.

### RalGAPα1 and RalA-GDP promote oligomerization of SERCA2a

**Fig. 5:**
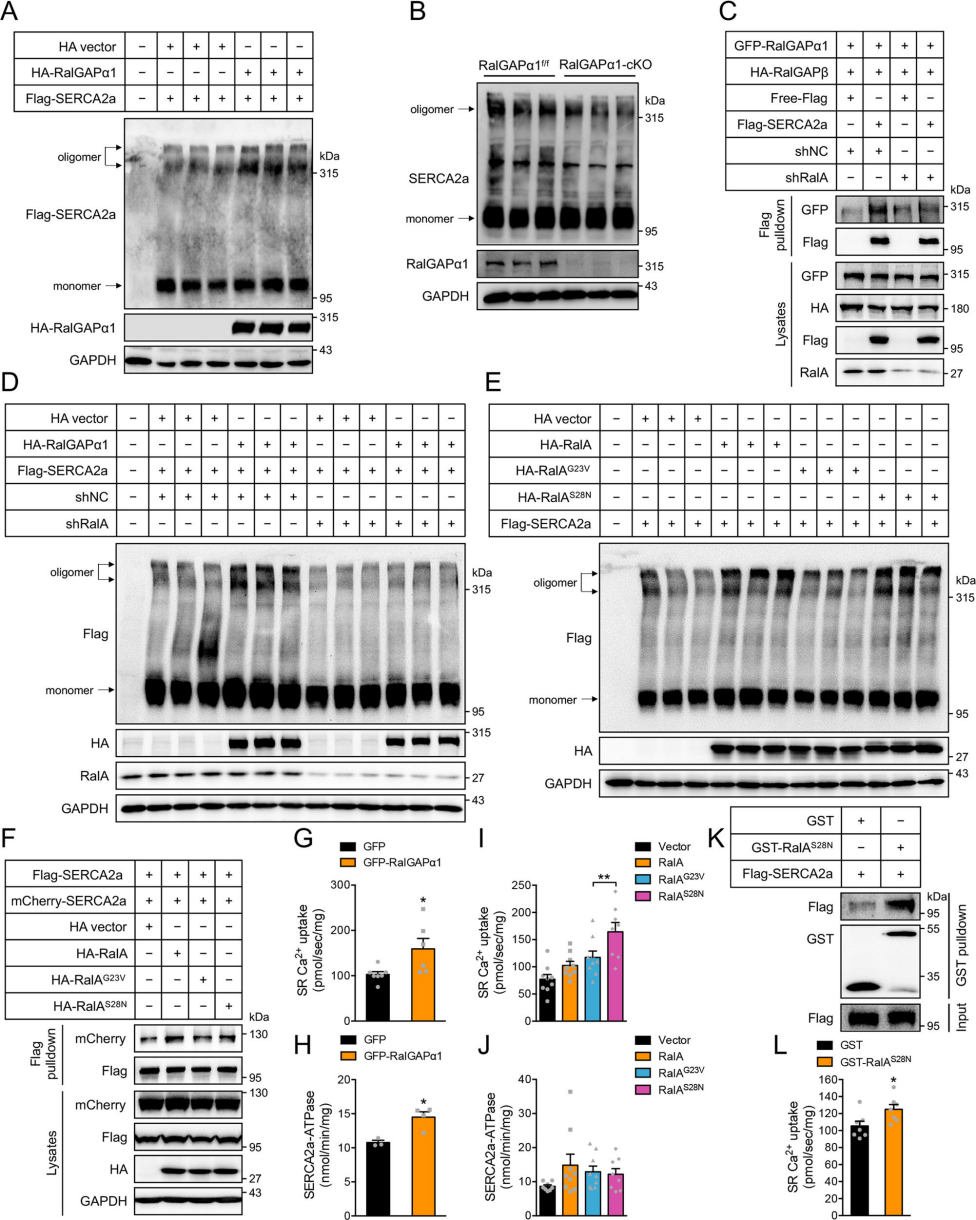
Regulation of SERCA2a oligomerization by the RalGAPα1-RalA axis. **A** Oligomerization of SERCA2a in HEK293 cells in which SERCA2a was co-expressed with RalGAPα1 or an empty vector. **B** Oligomerization of SERCA2a in the heart of RalGAPα1-cKO mice and RalGAPα1^f/f^ littermates (2-month-old, female). **C** GFP-RalGAPα1/HA-RalGAPβ complex was co-expressed with Flag-SERCA2a or free Flag in HEK293 cells that were infected with lentivirus-shNC or lentivirus-shRalA. After immunoprecipitation with the Flag antibody, GFP-RalGAPα1 was detected in the immunoprecipitates via western blot. **D** HA-RalGAPα1 or HA-vector was co-expressed with Flag-SERCA2a in HEK293 cells that were infected with lentivirus-shNC or lentivirus-shRalA. Oligomerization of Flag-SERCA2a was analyzed via western blot. **E** Oligomerization of SERCA2a in HEK293 cells in which SERCA2a was co-expressed with HA-RalA, HA-RalA^G23V^, HA-RalA^S28N^ or an empty vector. **F** Flag-SERCA2a and mCherry-SERCA2a were co-expressed with HA-RalA, HA-RalA^G23V^, HA-RalA^S28N^ or HA-vector in HEK293 cells. After immunoprecipitation with the Flag antibody, mCherry-SERCA2a was detected in the immunoprecipitates via western blot. **G**, **H** SERCA2a Ca^2+^-transporting activity **G** and ATPase activity **H** in microsomes isolated from HEK293 cells in which SERCA2a was co-expressed with RalGAPα1 or an empty vector. SERCA2a Ca^2+^-transporting activity: *n* = 7 (GFP) and 6 (GFP-RalGAPα1). SERCA2a ATPase activity: *n* = 3 (GFP) and 4 (GFP-RalGAPα1). *p* = 0.027 (SR Ca^2+^-uptake) and 0.013 (SERCA2a-ATPase). **I**, **J** SERCA2a Ca^2+^-transporting activity **I** and ATPase activity **J** in microsomes isolated from HEK293 cells in which SERCA2a was co-expressed with HA-RalA, HA-RalA^G23V^, HA-RalA^S28N^ or an empty vector. SR Ca^2+^-uptake: *n* = 9 (vector, HA-RalA and HA-RalA^G23V^) and 8 (HA-RalA^S28N^). SERCA2a-ATPase: *n* = 8 (vector and HA-RalA^S28N^) and 9 (HA-RalA and HA-RalA^G23V^). SR Ca^2+^-uptake: *p* = 0.358 (HA-RalA vs HA-RalA^G23V^) and 0.0075 (HA-RalA^G23V^ vs HA-RalA^S28N^). SERCA2a-ATPase: *p* = 0.504 (HA-RalA vs HA-RalA^G23V^) and 0.814 (HA-RalA^G23V^ vs HA-RalA^S28N^). **K** GST-pulldown of Flag-SERCA2a from HEK293 cells. Recombinant GST-RalA^S28N^ protein was used as a prey to pull down Flag-SERCA2a from HEK293 cells. **L** SERCA2a Ca^2+^-transporting activity in microsomes isolated from HEK293 cells expressing Flag-SERCA2a. Recombinant GST or GST-RalA^S28N^ proteins were added to microsomes before the assay. *n* = 7. *p* = 0.030. The data are given as the mean ± SEM. Statistical analyses were carried out using two-sided t-test for 5**G**, **H** and one-way ANOVA for 5**I**, **J**. One-asterisk indicates *p* < 0.05. Source data are provided as a Source Data file.

SERCA2a switches between monomers and oligomers, and its oligomerization enhances its activity for transporting Ca^2+ ^^24,25^. Interestingly, visualizing blots of SDS-gels revealed that oligomerization of SERCA2a was markedly increased when co-expressed with the RalGAPα1/β complex in HEK293 cells (Fig. 5A, Supplementary Fig. 10A). In agreement, oligomerization of SERCA2a was significantly decreased in the RalGAPα1-cKO heart (Fig. 5B, Supplementary Fig. 10B). This decrease of SERCA2a oligomerization was not due to changes in its Thr^484^ phosphorylation that was not altered in the RalGAPα1-cKO heart (Supplementary Fig. 2D). The effects of RalGAPα1 on SERCA2a interaction and oligomerization were mediated by the downstream target RalA. Interaction of RalGAPα1 with SERCA2a and oligomerization of SERCA2a induced by RalGAPα1 were both prevented in cells where RalA was knocked down via shRNA (Fig. 5C, D, Supplementary Fig. 10C). Expression of the RalA^S28N^ mutant, but not the RalA^G23V^ mutant, elevated levels of high molecular weight SERCA2a (~300 kDa) and promoted interaction between two SERCA2a monomers (Fig. 5E, F, Supplementary Fig. 10D). In contrast, neither the GTP-bound RalB^Q72L^ mutant nor the GDP-bound RalB^S28N^ mutant affected the oligomerization of SERCA2a (Supplementary Fig. 5E).

In agreement with impaired functions of SERCA2a in the RalGAPα1-cKO heart (Fig. 3J, K), overexpression of RalGAPα1 enhanced the ATPase and transport activities of this Ca^2+^ pump in HEK293 cells (Fig. 5G, H). Expression of the RalA^S28N^ mutant significantly increased the Ca^2+^ transport activity of SERCA2a in HEK293 cells, but did not affect its ATPase activity, as compared to the RalA^G23V^ mutant (Fig. 5I, J). We then investigated whether RalA exerted a direct effect on SERCA2a to regulate its Ca^2+^ transport activity. To this end, we expressed and purified a GST-RalA^S28N^ recombinant protein from *E. coli*, and found it could interact Flag-SERCA2a in a GST-pulldown assay (Fig. 5K). Importantly, this GST-RalA^S28N^ recombinant protein increased the Ca^2+^ transport activity of SERCA2a when added to microsomes purified from HEK293 cells expressing Flag-SERCA2a (Fig. 5L), suggesting that RalA directly activates SERCA2a for Ca^2+^ transport. Together, these data show that RalGAPα1 promotes SERCA2 oligomerization through GDP-bound RalA to enhance the transport activity of this Ca^2+^ pump. RalGAPα1 may also regulate the ATPase activity of SERCA2a through its direct interaction with the Ca^2+^ pump.

### Expression of the GDP-bound RalA^S28N^ mutant protects against TAC-induced cardiomyopathy

**Fig. 6:**
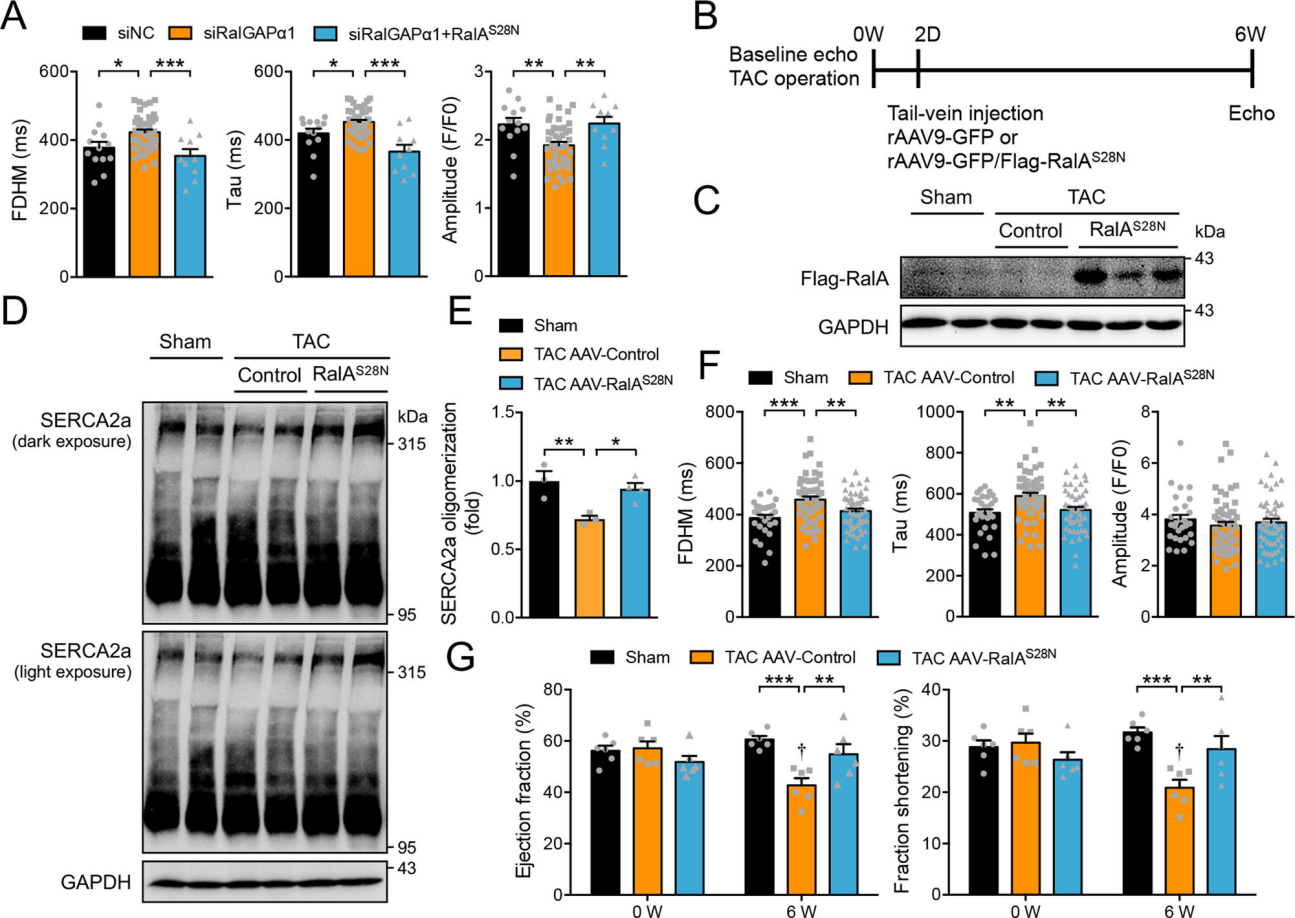
Effects of the GDP-bound RalAS28N protein on TAC-induced cardiomyopathy. **A** Ca^2+^ transients upon field stimulation in RalGAPα1-depleted neonatal rat cardiomyocytes that were transfected with or without RalA^S28N^. Amplitude, FDHM and Tau of Ca^2+^ transients were quantified from 13 (siNC), 44 (siRalGAPα1), and 10 (siRalGAPα1 + RalA^S28N^) cells. FDHM: *p* = 0.012 (siNC vs siRalGAPα1) and 0.0006 (siRalGAPα1 vs siRalGAPα1 + RalA^S28N^). Tau: *p* = 0.024 (siNC vs siRalGAPα1), and *p* < 0.0001 (siRalGAPα1 vs siRalGAPα1 + RalA^S28N^). Amplitude: *p* = 0.0041 (siNC vs siRalGAPα1) and 0.0071 (siRalGAPα1 vs siRalGAPα1 + RalA^S28N^). **B** Experimental design for AAV-mediated gene therapy of TAC-induced cardiomyopathy. Male C57BL/6 J mice (12-week-old) were subjected to TAC or sham surgery. AAV9-GFP or AAV9-RalA^S28N^ were administered into mice via vein injection on 2 days after surgery. Cardiac function was monitored before and after treatment. **C** Flag-RalA^S28N^ mutant protein expression in the heart of AAV9-GFP or AAV9-RalA^S28N^ administered male mice (20-week-old). **D**, **E** Oligomerization of SERCA2a in the heart of AAV9-GFP or AAV9-RalA^S28N^ administered male mice (20-week-old) that were subjected to TAC or sham surgery. **D**, representative blots. **E** quantitation of SERCA2a oligomerization. Data were presented as fold changes of the ratio of oligomer SERCA2a to total SERCA2a. *n* = 3 (Sham and TAC AAV9-Control) and 4 (TAC AAV9-RalA^S28N^). *p* = 0.0063 (Sham vs TAC AAV9-Control) and 0.014 (TAC AAV9-Control vs TAC AAV9-RalA^S28N^). **F** Ca^2+^ transients in primary cardiomyocytes isolated from AAV9-GFP or AAV9-RalA^S28N^ administered male mice (16-week-old) that were subjected to TAC or sham surgery upon field stimulation. Amplitudes, FDHM and Tau of Ca^2+^ transients were measured in 28 Sham cells, 54 TAC-AAV-Control cells and 51 TAC-AAV-RalA^S28N^ cells, respectively. FDHM: *p* = 0.0001 (Sham vs TAC AAV9-Control) and 0.0037 (TAC AAV9-Control vs TAC AAV9-RalA^S28N^). Tau: *p* = 0.0012 (Sham vs TAC AAV9-Control) and 0.0014 (TAC AAV9-Control vs TAC AAV9-RalA^S28N^). Amplitude: *p* = 0.314 (Sham vs TAC AAV9-Control) and 0.533 (TAC AAV9-Control vs TAC AAV9-RalA^S28N^). G. Cardiac function in AAV9-GFP or AAV9-RalA^S28N^ administered male mice that were subjected to TAC or sham surgery. *n* = 6. Ejection fraction: *p* = 0.793 (0 W/Sham vs 0 W/TAC AAV9-Control), *p* = 0.162 (0 W/TAC AAV9-Control vs 0 W/TAC AAV9-RalA^S28N^), *p* < 0.0001 (6 W/Sham vs 6 W/TAC AAV9-Control), *p* = 0.0005 (0 W/TAC AAV9-Control vs 6 W/TAC AAV9-Control), and *p* = 0.0027 (6 W/TAC AAV9-Control vs 6 W/TAC AAV9-RalA^S28N^). Fraction shortening: *p* = 0.718 (0 W/Sham vs 0 W/TAC AAV9-Control), *p* = 0.164 (0 W/TAC AAV9-Control vs 0 W/TAC AAV9-RalA^S28N^), *p* < 0.0001 (6 W/Sham vs 6 W/TAC AAV9-Control), *p* = 0.0008 (0 W/TAC AAV9-Control vs 6 W/TAC AAV9-Control), and *p* = 0.0034 (6 W/TAC AAV9-Control vs 6 W/TAC AAV9-RalA^S28N^). The data are given as the mean ± SEM. Statistical analyses were carried out using one-way ANOVA for **A**, **E** and **F**, and two-way ANOVA for **G**. One-asterisk indicates *p* < 0.05, two-asterisk indicates *p* < 0.01 and three-asterisk indicates *p* < 0.001. One-dagger indicates *p* < 0.001 (TAC AAV-control 0 W vs 6 W). Source data are provided as a Source Data file.

We next sought to find out whether elevation of the GDP-bound form of RalA might be of therapeutic value for treatment of pressure overload-induced cardiomyopathy. We first examined whether expression of RalA^S28N^ mutant would restore Ca^2+^ homeostasis in RalGAPα1-depleted NRVCs. Indeed, expression of RalA^S28N^ mutant protein reversed the RalGAPα1-depletion induced increases of FDHM and Tau, and relieved the depression of Ca^2+^ transient peaks resulted from RalGAPα1-deficiency in NRVCs (Fig. 6A). In contrast, expression of RalB^S28N^ had no such effects on Ca^2+^ transient (Supplementary Fig. 11A). As a proof-of-concept experiment, we then expressed the RalA^S28N^ mutant specifically in the heart using a Ctnt-promoter driven expression cassette in the AAV system. TAC surgery was conducted in WT mice, and a control AAV (rAAV9-GFP) or a RalA^S28N^-expressing AAV (rAAV9-GFP/Flag-RalA^S28N^) was then administered into mice through vein injection 2 days after surgery (Fig. 6B). We confirmed that the RalA^S28N^ mutant was expressed only in the heart but not in the other tissues analyzed including skeletal muscle and liver at both mRNA and protein levels (Fig. 6C, Supplementary Fig. 11B–D). The TAC surgery caused a decrease of SERCA2a oligomerization and prolonged SR Ca^2+^ re-uptake in cardiomyocytes, which were both prevented by infection with the AAV expressing the RalA^S28N^ mutant (Fig. 6D–F). In contrast, infection with the control AAV did not ameliorate cardiac dysfunction induced by the TAC surgery. Both EF and FS were significantly decreased in the TAC group infected with the control AAV as compared to the sham group at 6 weeks after surgery (Fig. 6G). Importantly, infection with the AAV expressing the RalA^S28N^ mutant prevented TAC-induced decline of EF and FS (Fig. 6G), showing that RalA^S28N^ expression helped to preserve cardiac function in pressure-overloaded mouse heart.

## Discussion

**Fig. 7:**
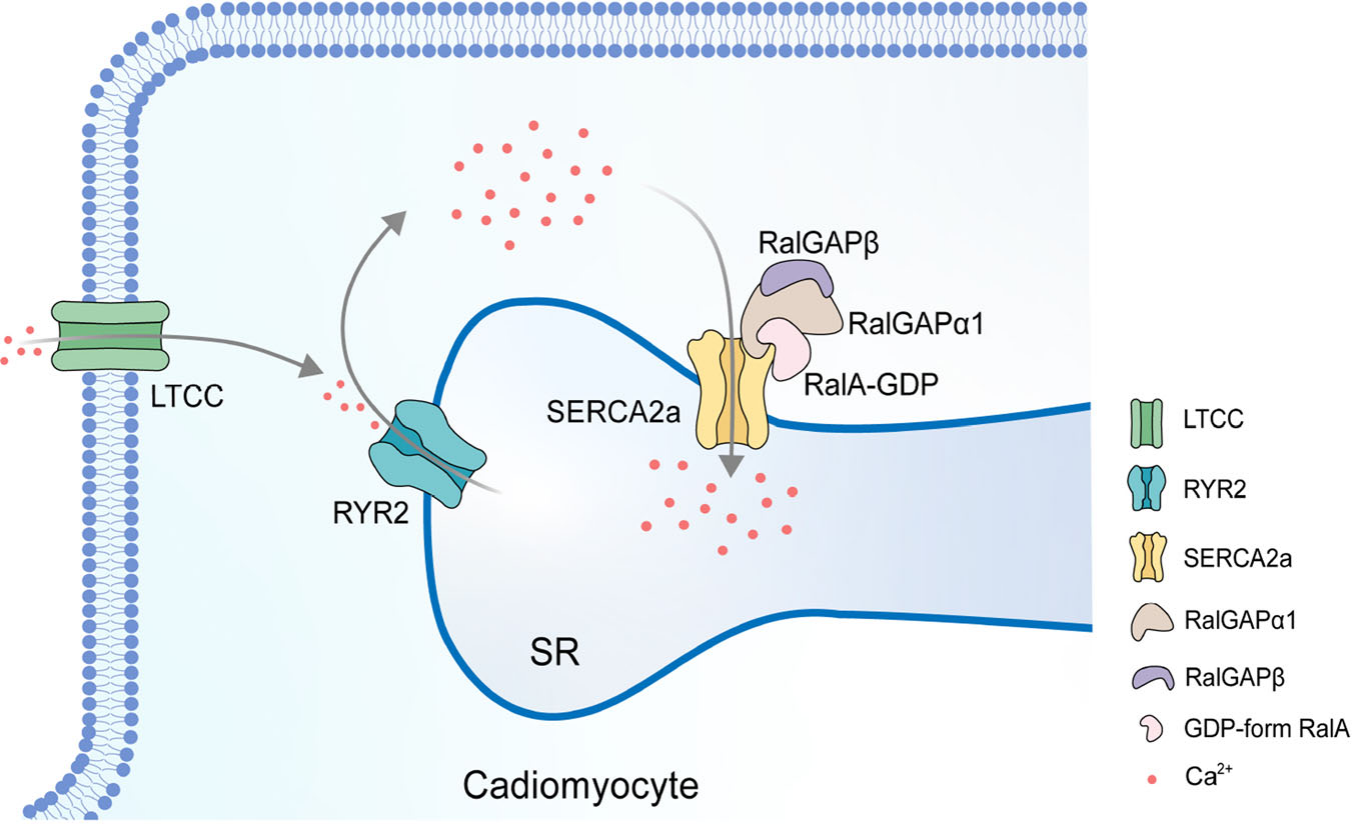
A model for the RalGAPα1−RalA signal module as a critical regulator of SERCA2a. A diagrammatic illustration represents the proposed model in which the RalGAPα1−RalA signal module regulates Ca^2+^ homeostasis in cardiomyocytes via interacting with SERCA2a. GDP-bound RalA interacts with SERCA2a, and enhances its function to regulate Ca^2+^ homeostasis. Pressure-overload upregulates RalGAPα1 in the heart and converts RalA into its GDP-bound form, which consequently plays a protective role in the heart.

Our findings show that the RalGAPα1 − RalA signal module is critical for protecting cardiac function. Based on these findings, we put forward a model in which RalGAPα1 interacts with SERCA2 and thereby regulates its activity and consequent SR Ca^2+^ re-uptake through the GDP-bound RalA (Fig. 7). Up-regulation of RalGAPα1 plays a protective role in pressure-overloaded hearts.

Phospholamban is a well-known regulator of SERCA2, which binds to the Ca^2+^ pump and imposes an inhibitory effect^6^. In contrast to the negative regulation by phospholamban, RalA exerts a positive regulation of SERCA2 upon binding to the Ca^2+^ pump. Both phospholamban and RalA are tightly regulated and exhibit dynamic interaction with SERCA2. Phospholamban is under the regulation of phosphorylation by protein kinase A, which dissociates phospholamban from SERCA2 and relieves its inhibitory effect on the Ca^2+^ pump^26^. Our findings show that guanine nucleotide-binding states dictate the dynamic interaction of RalA with SERCA2. Guanine nucleotide-binding states of RalA are controlled by its upstream regulators RalGAP and Ral guanine nucleotide exchange factors (RalGEFs)^11^. The upstream regulator RalGAPα1 also binds to SERCA2, however, it remains unclear whether this interaction is static or dynamic. There are seven RalGEFs in human cells^11^, and it is not known which RalGEF controls guanine nucleotide-binding states of RalA to regulate SERCA2. Besides these protein interactors, protein modifications such as phosphorylation and SUMOylation also regulate SERCA2^7,9^. For example, Thr^484^ phosphorylation of SERCA2 by SPEG promotes dimerization of SERCA2 and enhances its Ca^2+^ transport activity without affecting its ATPase activity^7^. RalA binding also induces SERCA2 dimerization, and increases its Ca^2+^ transport activity but not its ATPase activity. However, RalA binding might alter SERCA2 configuration to promote dimerization of the Ca^2+^ pump, which is not dependent on Thr^484^ phosphorylation. The complex regulation of SERCA2 manifests the importance of this Ca^2+^ pump in the heart. It is conceivable that certain coordination among these positive and negative regulations may exist to tightly control SERCA2 activity and thereby to adjust Ca^2+^ homeostasis to meet contractile demands under physiological and pathological conditions.

A general conception about small GTPases is that their GTP-bound forms are active and fulfill their functions through binding to its targets, whereas their GDP-bound forms are usually inactive^27^. However, accumulating evidence shows that Rab small GTPases can exert their functions in their GDP-bound forms. For example, GDP-bound Rab8a promotes fusion of lipid droplets in adipocytes and muscle cells^28,29^, and mediates secretion of insulin-like growth factor-1 in hepatocytes^30^. GDP-bound Rab27a controls endocytosis through interacting with coronin-3 in insulin-secreting cell lines^31^. In this study, we demonstrate that GDP-bound RalA interacts with SERCA2 and enhances its activity. As the list of functional GDP-bound small GTPases continues to expand, we may need to re-consider the well-accepted conception about GTP-bound small GTPases as their active forms. A systemic investigation to identify interactors of GTP-bound and GDP-bound small GTPases would help to find out whether both forms are active but display distinct functions.

The two RalGAP complexes display distinct expression patterns in skeletal muscle and adipose tissues, and are critical metabolic regulators in these two tissues^16–18^. They are both present in cardiomyocytes in the heart, and cardiac deletion of the RalGAPα1 causes no alteration of substrate oxidation in mitochondria and results in no energetic deficit. The presence of RalGAPα2 might be sufficient to maintain cardiac metabolism in the RalGAPα1-deficient heart. Clearly, the two RalGAP complexes are differentially regulated in the heart, and only RalGAPα1 responds to pressure overload in this tissue. This differential regulation of these two complexes suggests that they may have diverse functions in the heart or may regulate the same cellular processes but in response to different stimuli. It deserves a thorough investigation of possible roles that RalGAPα2 might play in regulation of cardiac metabolism and Ca^2+^ homeostasis in the future. The targets of these two RalGAP complexes, namely RalA and RalB, have overlapping roles with some specialized functions in certain cellular processes. For instance, RalA is required for anchorage-independent proliferation, whereas RalB is indispensable for survival of tumour cells^32^. Here, we show that RalA, but not RalB, interacts with SERCA2 and regulates SR Ca^2+^ re-uptake in its GDP-bound form. Thus, the RalGAP-Ral signaling modules may fulfill their diverse functions through different combinations. Potential subcellular compartmentation of these signaling modules as well as other regulatory factors such as RalGEFs can further increase the complexity, which ensures both specificities and robustness of signal networks.

SERCA2 dysfunction is a hallmark of heart failure, and restoration of SERCA2 function is an attractive strategy to treat this disease. AAV-mediated gene transfer of SERCA2 improves cardiac function in a swine model of heart failure secondary to volume overloading^33^ and in a sheep model of heart failure induced by rapid pacing^34^. Post-translational modifications are critical for SERCA2 activity and provide alternative ways to restore SERCA2 function for treatment of heart failure. SUMOylation is a critical modification of SERCA2, and both SUMO1 and SERCA2-SUMOylation are greatly decreased in failing hearts^9^. AAV-mediated gene transfer of SUMO1 improves cardiac function in a murine as well as a swine model of heart failure^9,35^. Moreover, activation of SERCA2 SUMOylation via a small molecule preserves ventricular function in mice with heart failure^36^. Modulation of SERCA2 through its interacting proteins is another potential strategy to improve cardiac function in failing hearts. Our proof-of-principle experiments with the RalA^S28N^ mutant demonstrate that this might be a promising therapeutic agent to treat heart failure.

In summary, we show that the RalGAPα1 − RalA signal module is critical for Ca^2+^ homeostasis and cardiac function through regulating SERCA2, and plays a protective role in failing hearts induced by pressure-overload. Our findings have therapeutic implications for treatment of heart failure secondary to pressure-overload.

## Methods

This study was carried out under approval of the Ethics Committee of Nanjing University complying with all relevant ethical regulations. All animal procedures involving mice and rats in this study were approved by the Institutional Animal Care and Use Committee (IACUC) at Model Animal Research Center of Nanjing University.

### Materials

Protein G-Sepharose was purchased from GE Healthcare (Little Chalfont, Buckinghamshire, UK). Precast NuPAGE® Bis-Tris gels were from Thermo Fisher Scientific (Waltham, MA, USA). NE and AngII were bought from MedChemExpress (Shanghai, China). All other chemicals were from Sigma-Aldrich (Shanghai, China) or Sangon Biotech (Shanghai, China). The commercial primary antibodies used in this study are listed in Supplementary Table 1, and used at a dilution of 1:1000 for immunoblotting. The RalGAPα1, RalGAPα2 and RalGAPβ antibodies were described previously^14^, and used at a dilution of 1:1000 for immunoblotting. The antibody recognizing pThr^484^-SERCA2a was as previously reported^7^, and used at 1 µg/ml for immunoblotting. Horseradish peroxidase (HRP)-conjugated goat anti-rabbit IgG (Cat No. 111-035-003), mouse anti-rabbit IgG (211-002-171), goat anti-mouse IgG (Cat No. 115-035-003) and goat anti-mouse IgG (115-005-174) were from Jackson ImmunoResearch Labs, and used at a dilution of 1:5000. Plasmids for RalGAPα1, RalA and SERCA2a were reported previously^7,16^.

### Mouse breeding and husbandry

Mice and rats were housed under specific pathogen free conditions with a light/dark cycle of 12 h, ambient temperature 23 ± 2 °C and humidity 40–70%. Unless stated, mice and rats had free access to food and water in their home cages.

The RalGAPα1^f/f^ mouse was previously reported^17^, and used to mate with the αMHC-Cre mouse^20^ for generation of cardiomyocyte-specific RalGAPα1 knockout (RalGAPα1-cKO) mice on a C57BL/6 J background. RalGAPα1^f/f^ X RalGAPα1^f/f^-Cre mating was set up to generate RalGAPα1^f/f^ (control mice) and RalGAPα1^f/f^-Cre (RalGAPα1-cKO mice). The RalGAPα1^f/f^ mice were genotyped using the following primers: 5’-GAGATGGCGCAACGCAATTAATG-3’ and 5’-GGCTGCAAAGAGTAGGTAAAGTGCC-3’. Genotyping of the Cre mice were performed using the following primers: 5’-GCCTGCATTACCGGTCGATGC-3’ and 5’-CAGGGTGTTATAAGCAATCCC-3’.

Sprague-Dawley rats were mated to obtain neonatal pups for isolation of NRVCs.

### Oral glucose tolerance test

Oral glucose tolerance test was performed in mice that were restricted from food access overnight (16 h). After food deprivation, mice were administered with a bolus of glucose (1.5 mg/g) via oral gavage, and their blood glucose was measured through tail bleeding using a Contour-TS glucometer (Bayer).

### Echocardiography (Echo)

Echo analysis was performed in mice that were anaesthetized with gaseous isoflurane. Anaesthetized mice were analyzed via a Vevo 770 high-resolution in vivo micro-imaging system (VisualSonics, inc) with a 30 MHz RMV-707B ultrasonic probe. Left ventricle anterior wall (LVAW), left ventricle posterior wall (LVPW), left ventricle internal dimension (LVID), and left ventricle volume (LV Vol) of systole and diastole were determined on M-mode images. Equations for calculation of ejection fraction (EF) and fractional shortening (FS) are as follows, EF% = [(LV Vol;d - LV Vol;s)/LV Vol;d] x 100%, and FS% = [(LVID;d – LVID;s)/LVID;d] x 100%.

### TAC surgery

TAC surgery was performed on male mice (2-3-month-old) that were anaesthetized by intraperitoneal injection of ketamine (100 mg/kg) and xylazine (10 mg/kg). The aortic arch was surgically exposed and ligated with a 6-0 suture using a 27-gauge needle to yield a constriction of 0.413 mm in diameter. For sham operation, mice were subjected to the same surgical procedure only without ligation.

### Tissue homogenization and lysis

After harvest, mouse tissues were snap-frozen in liquid nitrogen, and stored at −80 °C before lysis. Tissues were homogenized in a lysis buffer (50 mM Tris-HCl (pH 7.4), 1 mM EDTA, 1 mM EGTA, 1% (v/v) Triton X-100, 1 mM sodium *ortho-*vanadate, 10 mM sodium glycerophosphate, 50 mM sodium fluoride 5 mM sodium pyrophosphate, 0.27 M sucrose, 2 µM microcystin-LR, 1 mM benzamidine, 0.1% (v/v) 2-mercaptoethanol, 0.2 mM phenylmethanesulfonyl fluoride, 1 mg/ml leupeptin, 1 mg/ml pepstatin and 1 mg/ml aprotinin). Tissue homogenates were lysed on ice for 30 min, and tissue debris was removed through centrifuge to obtain tissue lysates. Measurements of protein concentrations were carried out using Bradford reagent (Thermo Fisher Scientific).

### Immunoprecipitation and immunoblotting

Immunoprecipitation of target proteins was carried out through incubation of tissue or cell lysates with antibody-coupled protein G-Sepharose or GFP-binder (ChromoTek GmbH, Planegg-Martinsried, Germany) for 16 h at 4^o^C. Resins were washed to remove non-specific binding proteins, and immunoprecipitated protein complexes were then eluted in SDS sample buffer.

Lysates or immunoprecipitates were subjected to electrophoretical separation via SDS-PAGE. After electrophoresis, separated proteins were immunoblotted onto nitrocellulose membranes that were incubated with primary antibodies. After further probing with horseradish peroxidase (HRP)-conjugated secondary antibodies, membranes were incubated with a HRP substrate for enhanced chemiluminescence. Chemiluminescent signals were detected using a gel documentation system (Tanon, China), and quantified using Image J. Signal intensities of proteins of interest were presented as fold changes after normalization with loading controls. SERCA2a oligomerization was presented as fold changes of the ratio of oligomer SERCA2a to total SERCA2a (sum of monomer and oligomer SERCA2a).

### In vitro pulldown assay

The recombinant proteins GST-RalA, GST-RalA^G23V^, GST-RalA^S28N^, and MBP-RalGAPα1 were expressed and purified from *E. coli*. Flag-RalGAPα1 was expressed in HEK293 cells, captured with the Flag beads and eluted using the Flag peptide. GST-RalA was loaded with 2 mM GDP or 200 μM GTPγS in a loading buffer (20 mM Tris-Cl (pH=7.4), 1 mM DTT, 50 mM NaCl, 2 mM EDTA,0.2 mM PMSF, 1 μg/ml leupeptin, 1 mM benzamidine, 1 mM aprotinin, 1 mM pepstatin) for 1 h at 25 °C. GST-RalA^G23V^ was loaded with 200 μM GTPγS, and GST-RalA^S28N^ was loaded with GDP for 1 h at 25 °C. Afterwards, MgCl_2_ (10 mM) was added to stop loading. The GDP or GTPγS loaded GST-RalA, GST-RalA^G23V^ and GST-RalA^S28N^ were then coupled to glutathione beads (GE Healthcare). GST was also bound to glutathione beads and used as a control. The uncoupled proteins were removed, and the beads were washed with the loading buffer for twice. The GST recombinant protein-loaded beads were incubated with MBP-RalGAPα1 or Flag-RalGAPα1 overnight at 4 °C. After incubation, the glutathione beads were washed five times in a buffer (25 mM Tris, pH 7.5, 40 mM NaCl, 30 mM MgCl_2_, 1% NP-40, and 1 mM DTT). The proteins were eluted into the sample buffer at 95 °C and subjected to SDS-PAGE.

### Mass-spectrometry

Immunoprecipitated protein complexes were separated via SDS-PAGE and visualized by Commassie blue staining. Then, protein bands of interest were excised from gels and subjected to in-gel digestion using trypsin as the digestion enzyme. Resultant peptides of each gel band were analyzed by LC-MS/MS. Briefly, MS data acquisition was performed with a NanoLC.2D (Eksigent Technologies) coupled with a TripleTOF 5600+ System (AB SCIEX). Original MS/MS data were submitted to ProteinPilot Software (version 4.5, AB Sciex) for data analysis and searched against UniProt database to identify peptides and proteins.

### AAV9-mediated cardiomyocyte-specific gene expression in mice

The recombinant adeno-associated virus serotype-9 (AAV9) was used for gene delivery in mice as previously described with modifications^37^. The expression of Flag-RalA^S28N^ or Flag-RalA^G23V^ was under control of a cardiac-specific Ctnt promoter. The recombinant AAV9 was administered into mice via tail vein injection at 4 × 10^11^ vector genomes (vg) per mouse.

### Cell culture and transfection

Human embryonic kidney HEK293 cells were bought from the Cell Resource Center, Chinese Academy of Medical Sciences and Peking Union Medical College (China), and maintained in DMEM medium containing 10% (v/v) foetal bovine serum with regular tests of mycoplasma. Cells were transfected using Lipofectamine 3000 reagent (Thermo Fisher Scientific).

### Isolation of primary mouse cardiomyocytes

A collagenase-based method was used to isolate primary mouse cardiomyocytes with modifications^38^. Briefly, hearts of heparin-treated mice were perfused with a collagenase solution (1 mg/ml) using a Langendorff system (ADInstruments). After collagenase digestion, cell suspensions were passed through a cell strainer (100 μm of mesh size) to remove tissue debris. Resultant cardiomyocytes were rinsed for three times in Krebs-Henseleit buffer B that contains 5 mM taurine and 10 mM 2,3-butanedione monoximine with increasing Ca^2+^ (1^st^ wash: 0.1 mM, 2^nd^ wash: 0.2 mM, and 3^rd^ wash: 0.6 mM).

### Isolation and transfection of primary neonatal rat cardiomyocytes

Primary neonatal rat cardiomyocytes were obtained from ventricles of neonatal Sprague Dawley rats (postnatal day 0–3). Briefly, ventricles were isolated from neonatal rats and minced into cubes. Ventricle cubes were subjected to sequential digestion with trypsin (0.25%) at 4 °C overnight and collagenase (1 mg/ml) at 37 °C for 15 min. Cell suspensions were filtered through a cell strainer (70 μm of mesh size) to remove tissue debris. Resultant cells were seeded in DMEM containing 10% (v/v) foetal bovine serum for 1 h to allow fibroblasts to settle down for removal. Afterwards, cardiomyocytes were plated in fresh DMEM supplemented with 10% (v/v) foetal bovine serum. Transfection of primary neonatal rat cardiomyocytes was carried out using Lipofectamine 3000 reagent (Thermo Fisher Scientific). The sequences of siRNAs used in this study are as follows. siRalGAPα1: 5′-GCACAAAGGGAAAGGAGUUUU-3′. siRalA: 5′-GCAGACAGCUACAGGAAGAUU-3′. siRalB: 5′-GGACAAGGTGTTCTTTGAT-3′. shRalA: 5′-GCTAGAAGTCTGTCCAAATTT-3’.

### Calcium transients in primary cardiomyocytes

A Fluo-4-AM based method was used to measure calcium transients in cardiomyocytes^39^. Briefly, cardiomyocytes were incubated in Hanks buffer containing 1 mM MgCl_2_, 1 mM CaCl_2_, 2% (w/v) BSA, and 5 μM Fluo-4-AM (Thermo Fisher Scientific). After loaded with Fluo-4-AM, cardiomyocytes were subjected to electrical stimulation with a GRASS S48 stimulator (frequency 0.5 Hz, duration 60 ms, decay 40 ms, voltage 80 V, repeat). Line-scan images were taken using a Zeiss LSM880 confocal microscope, and analyzed with IDL5.5 (Harris Geospatial Solutions). The time elapsing from the peak of calcium transients to 63% from the peak to the basal level in the fading phase was defined as the decay time (Tau).

### Calcium imaging in HEK293 cells

HEK293 cells expressing SERCA2a were loaded with 5 μM Fluo-4-AM, and then stimulated with 100 μM ATP. Frame scan images of cells were taken using an Olympus confocal microscope for ~270 s.

### Imaging and analysis of t-tubules (TT) in cardiomyocytes

A Di-8-ANEPPS based imaging method was used to analyze TT organization in cardiomyocytes^40^. Primary cardiomyocytes were isolated and stained with Di-8-ANEPPS (10 μM). Cell images were obtained using a Carl Zeiss 880 confocal microscope, and subjected to Fast Fourier Transformation for analysis of TT organization. TT power (the peak amplitude) in the Fourier spectrum of cell images was determined using ImageJ software (version 1.46) with a plugin TTorg (http://mirror.imagej.net/plugins/ttorg).

### Measurements of SERCA2 ATPase activity in microsomes

Microsomes containing crude SR membrane vesicles were isolated for measurement of the ATPase activity of SERCA2^41^. ATP hydrolysis reaction was performed via incubating microsomes (50 μg protein) with an assay buffer containing 100 mM KCl, 10 mM HEPES (pH 7.4), 5 mM MgCl_2_, 100 μM CaCl_2_, 1.5 mM ATP, 2 μM A23187 and 5 mM sodium azide at 30 °C for 30 min, and terminated by addition of ice-cold 10% TCA. The reactions were carried out in the absence or presence of 5 μM thapsigargin to determine total or thapsigargin-insensitive calcium pumps, respectively. The activity of thapsigargin-sensitive Ca^2+^-ATPase (SERCA2-ATPase) was calculated through subtraction of the thapsigargin-insensitive Ca^2+^-ATPase activity from total activity.

### Measurements of Ca^2+^ uptake in microsomes

Microsomes containing crude SR membrane vesicles were isolated and used to measure Ca^2+^ uptake with a Fura-2 based method^42^. Briefly, microsomes were resuspended in an assay buffer (100 mM KCl, 10 mM HEPES-KOH (pH 7.4), 10 mM oxalate, 5 mM MgCl_2_, and 10 μM ruthenium red) containing 2 μM Fura-2 free acid. ATP (5 mM) and Ca^2+^ (2 μM) were added to initiate the uptake of Fura-2 into microsomes. Dual excitation at 340 and 380 nM, respectively, was performed, and the emitted fluorescence was recorded at 510 nM using a fluorescence microplate reader (BioTek). Calculation of free Ca^2+^ in the microplate was carried out using the equation, ${{{{{{{\rm{free}}}}}}}}\ {{{{{{{\rm{calcium}}}}}}}}={{{{{{{\rm{Kd}}}}}}}}\times {{\upbeta}}\times \frac{\left(R-R{\min }\right)}{\left(R{\max}-R\right)}$^43^. Free Ca^2+^ was plotted against the assay duration using Clampfit 10.4 (Molecular Devices). Ca^2+^ uptake rates were calculated using the linear portion of the curve after initiation of the uptake reaction.

### Masson’s staining

Mice were sacrificed for isolation of hearts. Then, hearts were fixed in 4% PFA overnight at 4 °C, and embedded in paraffin wax. Afterwards, hearts were sectioned into 5-μm-thick slices using a Leica RM2016 microtome. Heart slices were subjected to sequential staining with Biebrich scarlet for 50 sec, phosphotungstic acid and phosphomolybdic acid for 10 min, and Aniline blue for 4 min. The stained sections were photographed using an Olympus BX53F microscope.

### RNA isolation and quantitative PCR (QPCR)

Total RNA was extracted from tissues or cells using the TRIzol® Reagent (Life Technologies), and reverse-transcribed into cDNAs using a PrimeScript® RT reagent kit (DRR047A, TaKaRa). QPCR analyses of genes of interest were carried out using a Roche Lightcycler Real-Time PCR system with primers listed in Supplementary Table 2.

### Statistics and reproducibility

Data analyses were performed via *t*-test for two groups, or via one-way or two-way ANOVA for multiple groups using Prism software (GraphPad, San Diego, CA, USA). Differences were considered statistically significant at *p* < 0.05.

Except for mass-spec experiments, similar results were obtained from at least two experiments.

### Reporting summary

Further information on research design is available in the Nature Research Reporting Summary linked to this article.

## Supplementary information

Updated Supplementary file

Description of additional supplementary items

Supplementary Data 1

Reporting Summary

## Data Availability

All data generated or analysed during this study are included in this published article and its supplementary information files. Proteomics data have been deposited in the MassIVE Repository in University of California, San Diego (ftp://massive.ucsd.edu/MSV000089597). Source data are provided with this paper.

## Source data

source data
