# Author Correction: The RalGAPα1–RalA signal module protects cardiac function through regulating calcium homeostasis

**DOI:** 10.1038/s41467-022-33574-5

**Published:** 2022-10-10

**Authors:** Sangsang Zhu, Chao Quan, Ruizhen Wang, Derong Liang, Shu Su, Ping Rong, Kun Zhou, Xinyu Yang, Qiaoli Chen, Min Li, Qian Du, Jingzi Zhang, Lei Fang, Hong-Yu Wang, Shuai Chen

**Affiliations:** 1 State Key Laboratory of Pharmaceutical Biotechnology and MOE Key Laboratory of Model Animal for Disease Study, Department of Cardiology, Nanjing Drum Tower Hospital, The Affiliated Hospital of Nanjing University Medical School, Model Animal Research Center, Nanjing University, Nanjing, China; 2 School of Medicine, Nanjing University, Nanjing, China

**Keywords:** Heart failure, Calcium signalling, Preclinical research

**Correction to**: *Nature Communications* 10.1038/s41467-022-31992-z, published online 25 July 2022

The original version of this article contained an error in Supplementary Fig. 3B, in which the unit for ATP was reported as μmol/μg protein, while it should have been ‘nmol/mg protein. The HTML has been updated to include a corrected version of the Supplementary Information.

## Supplementary information

updated Supplementary file

