# Supplementary material for: The RalGAPα1–RalA signal module protects cardiac function through regulating calcium homeostasis
Source: Nat Commun. 2022 Jul 25;13:4278. doi: 10.1038/s41467-022-31992-z (PMC9314365; doi:10.1038/s41467-022-31992-z)

Supplementary Information for

The RalGAPα1−RalA signal module protects cardiac function through regulating calcium homeostasis

Zhu et al.

Supplementary Table 1 The list of commercial antibodies used in this study

Antibody Name	Company	Cat No.
anti-RalA	CST	#4799
anti-RalB	Abclonal	WH079944
anti-β-MHC	Sigma	t9283
anti-cTnT	Thermo Fisher	MA5-12960
anti-DDR2	Santa Cruz Biotechnology	sc-8989
anti-SERCA2a	Proteintech	13985-1-AP
anti-pT202/Y204-ERK1/2	CST	4370
anti-ERK1/2	CST	4695
anti-pT180/Y182-p38	CST	4511
anti-p38	CST	8690
anti-pS473-PKB	CST	9271S
anti-PKB	CST	9272S
anti-RalGAPa1	Invitrogen	PA5-51467
anti-PDI	CST	3501S
anti-mCherry	Proteintech	26765-1-AP
anti-HA	CST	#3724
anti-Flag	Sigma	F9291
anti-GFP	Santa Cruz Biotechnology	sc-9996
anti-GST	Abclonal	AE001
anti-GAPDH	Proteintech	60004-1

Supplementary Table 2 QPCR Primers for analysis of target genes

Gene name	Forward primer	Reverse primer
RalGAPα1-Mus	5′-AGATCAGACGGGAAGGTGTT-3′	5′-CTTGAAGGCTGAGTGGAGGA-3′
RalGAPα2-Mus	5′-CAGGAGTGGAGAAGGCAAGA-3′	5′-TGGGGCTGTAACCTTGAGAG-3′
RalGAPβ-Mus	5‵-AAATCCAAGGAGCCACTGGA-3′	5′-GGCTCCAACTGCTTATTCCG-3′
RalGAPα1-Rat	5′-CCAGCACCACTTAGAGCCAA-3′	5′-GCGGCTTTTGCTAGTTCGAG-3′
RalGAPα2-Rat	5′-GTTCAGGTGAAATGGATCCTGC-3′	5′-TTCGGATGAGACCTCCTTGG-3′
RalGAPβ-Rat	5′-CCAGCTTATTTATCCAGCGTTATTC-3′	5′-ATGAGGGAGGGGCAACAAAG-3′
RalA	5′-ACAGGATGGCTGCAAACAAG-3′	5′-TGAACTGCAGAGTCAGAGCA-3′
RalB	5′-CTTTCCCTCCTCAACACCCT-3′	5′-AGCCTTCCCTTCATCTGCTT-3′
β-MHC	5‵-ACCCCTACGATTATGCG-3′	5‵-GTGACGTACTCGTTGCC-3′
Rcan1.4	5‵-GTGTGGCAAACGATGATGTC-3′	5‵-AGGAACTCGGTCTTGTGCAG3′
ANP	5′-TCGTCTTGGCCTTTTGGCT-3′	5′-TCCAGGTGGTCTAGCAGGTTCT-3′
BNP	5′-AAGCTGCTGGAGCTGATAAGA-3′	5′-GTTACAGCCCAAACGACTGAC-3′
Col1A1	5′-GGAGAGAGCATGACCGATGG-3′	5′-AAGTTCCGGTGTGACTCGTG-3′
Col3A1	5′-CCCAGAACATTACATACCA-3′	5′-GATTAAAACAAGATGAACAC-3′
Serca2a	5′-ACTTCTTGATCCTCTACGTG-3′	5′-AAATGGTTTAGGAAGCGGTT-3′
Pmca1	5′-TTAGTCTGGGAAGCATTACAAGATGTCAC-3′	5′-CTTCTTCCCCAACAGAAACTTCTCC-3′
Pmca4	5′-ACGTCTTCCCACCCAAGGTTC-3′	5′-CCAGCAGCCCACACTCTGTC-3′
Ncx	5′-GATCATCCGATTCCCTCTACTG-3′	5′-GTCAGTGGCTGCTTGTCATC-3′
Ryr2	5′-TCAAACCACGAACACATTGAGG-3′	5′-AGGCGGTAAAACATGATGTCAG-3′
Ltcc	5′-CAATGGTCAATGAAAACACGA-3′	5′-GGCTCCCATAGTTGGAACCT-3′
RalA-AAV	5′-TACGATGAGTTTGTGGAGGACT-3′	5′-CTCCTGCCCAGCTGTATCTAAG-3′
Rgl1	5′-GGAGCTGTTTACCATGTCACC-3′	5′-CTTGCAGGTCTCGTACTGACT-3′
Rgl2	5′-TTCTGTGTCAGTCTGGGACGA-3′	5′-GCCGGAGTGAACATCATGTCA-3′
Rgl3	5′-CGACACCTTCCTTCATTACCG-3′	5′-GGGGGTAGCAGAAAACCCA-3′
Ralgds	5′-CCTTCTTGGATCGTTGTCAATGA-3′	5′-CGTCTTCGGTCTGCCCATA-3′
Ralgds1	5′-TACCCGGAGGTTCAACCAG-3′	5′-GCACTTTGTAATGCTGACACCA-3′
Ralgds2	5′-GAAACCCACAGTAGCATCTTGG-3′	5′-CCGTTCACCTTTCTCGGAGT-3′
36B4-Mus	5′-TAAAGACTGGAGACAAGGTG-3′	5′-GTGTACTCAGTCTCCACAGA-3′
36B4-Rat	5′-TCCAGAGGTACCATTGAAATCC-3′	5′-GTAGATGCTGCCATTGTCAAAC-3′

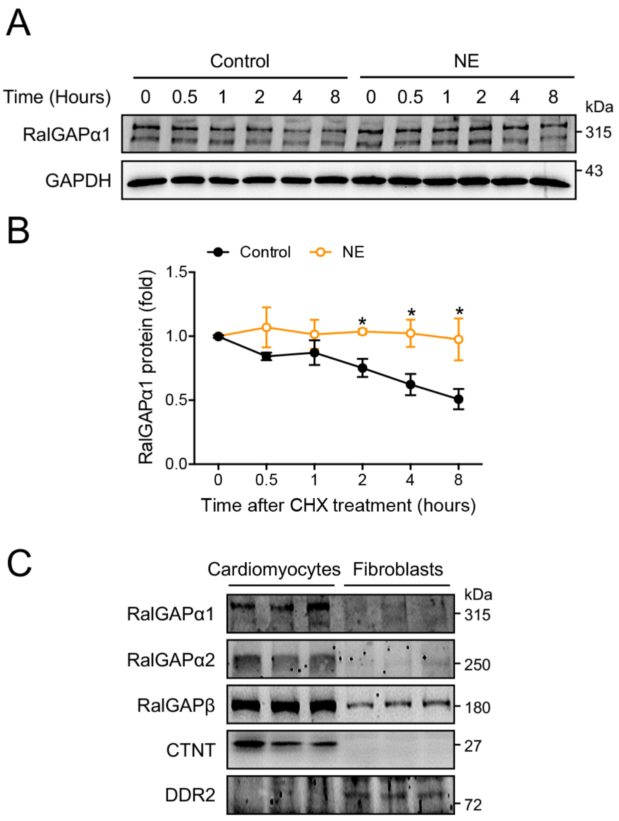

Supplementary Figure 1 RalGAPα1 protein expression and turnover in cardiomyocytes

A-B. Primary neonatal rat ventricular cardiomyocytes (NRVCs) were stimulated with or without norepinephrine (NE), and then treated with cycloheximide (CHX) for the indicated time. RalGAPα1 protein levels were determined via immunoblotting. A, representative blots. B. Quantitative data. n = 3 (Control/0.5 h, NE/2 h) and 4 (Control/0 h, 1 h, 2 h, 4 h, 6 h, and NE/0 h, 0.5 h, 1 h, 4 h, 6 h). $p = 0.913$ (0 h), 0.278 (0.5 h), 0.376 (1 h), 0.020 (2 h), 0.025 (4 h), and 0.043 (8 h).

C. Protein expression of RalGAPα1, RalGAPα2, and RalGAPβ in primary cardiomyocytes and fibroblasts isolated from the hearts of 2-month-old male mice. CTNT was used as a cardiomyocyte marker and DDR2 as a fibroblast marker.

The data are given as the mean ± SEM. Statistical analysis was carried out via two-sided t-test. One-asterisk indicates $p < 0.05$. Source data are provided as a Source Data file.

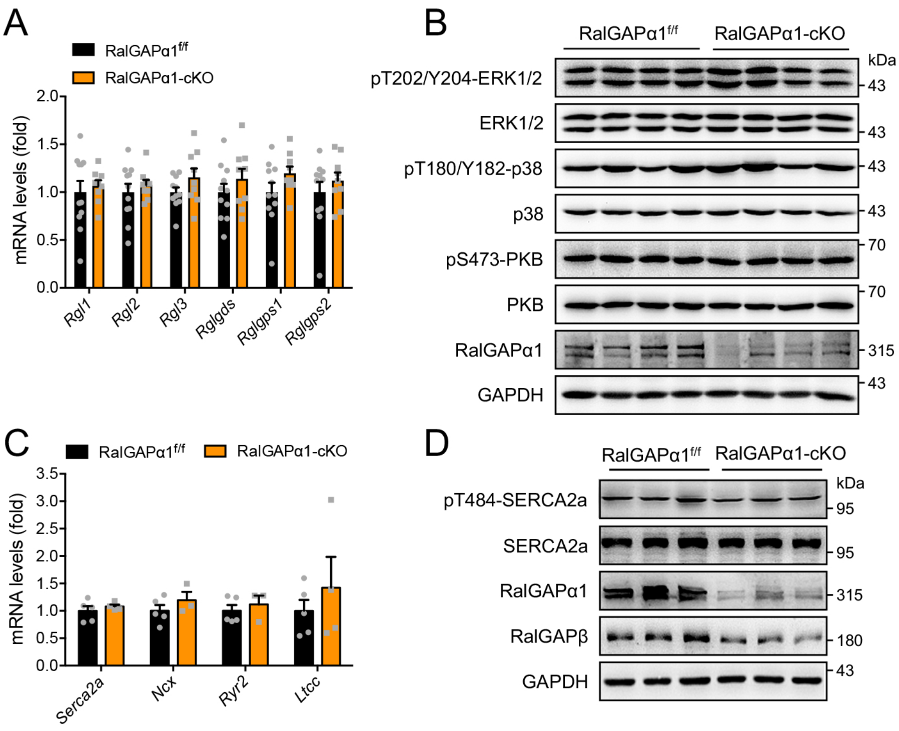

Supplementary Figure 2 Key regulators for cellular signaling and Ca^{2+} homeostasis in the heart of RalGAPα1-cKO mice

A. mRNA expression of RalGEFs in the hearts of male RalGAPα1-cKO and RalGAPα1$^{f/f}$ mice (3-month-old). n = 11 (RalGAPα1$^{f/f}$) and 9 (RalGAPα1-cKO). p = 0.642 (*Rgl1*), 0.496 (*Rgl2*), 0.145 (*Rgl3*), 0.314 (*Rglgds*), 0.138 (*Rglgps1*), and 0.410 (*Rglgps2*).

B. Expression and phosphorylation of ERK1/2, p38 and PKB in the hearts of male RalGAPα1-cKO and RalGAPα1$^{f/f}$ mice (4-month-old).

C. mRNA expression of key regulators for Ca^{2+} homeostasis in the hearts of male RalGAPα1-cKO and RalGAPα1$^{f/f}$ mice (2-month-old). RalGAPα1$^{f/f}$: n = 5. RalGAPα1-cKO: n = 4 (*Serca2a* and *Ltcc*) and 3 (*Ncx* and *Ryr2*). p = 0.462 (*Serca2a*), 0.317 (*Ncx*), 0.539 (*Ryr2*), and 0.463 (*Ltcc*).

D. Phosphorylation and expression of SERCA2a in the hearts of male RalGAPα1-cKO and RalGAPα1$^{f/f}$ mice (4-month-old).

The data are given as the mean ± SEM. Statistical analyses were carried out using two-sided t-test. Source data are provided as a Source Data file.

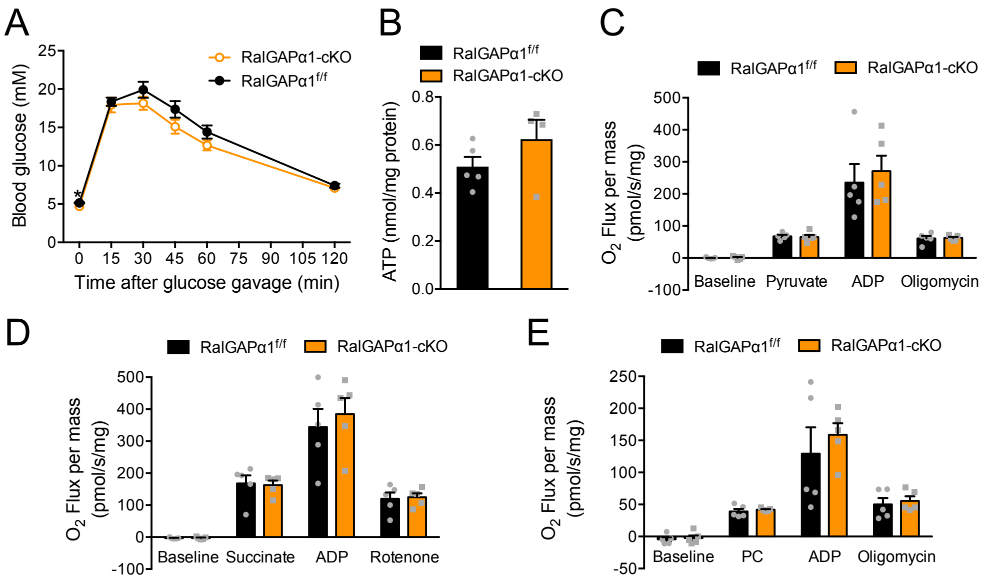

Supplementary Figure 3 Cardiac metabolism in the RalGAPα1-cKO heart

A. Oral glucose tolerance test in the male RalGAPα1-cKO and RalGAPα1$^{f/f}$ mice (2-month-old). The values show glucose area under the curve during glucose tolerance test. n = 7 (RalGAPα1$^{f/f}$) and 8 (RalGAPα1-cKO). $p = 0.015$ (0 min), 0.731 (15 min), 0.216 (30 min), 0.129 (45 min), 0.122 (60 min), and 0.392 (120 min).

B. ATP levels in hearts of the male RalGAPα1-cKO and RalGAPα1$^{f/f}$ mice (3-month-old). n = 5 (RalGAPα1$^{f/f}$) and 4 (RalGAPα1-cKO). $p = 0.217$.

C-E. Mitochondrial respiration rates in hearts of male RalGAPα1-cKO and RalGAPα1$^{f/f}$ mice (3-month-old). Heart tissue chunks were used to measure mitochondrial respiration rates using O2K with pyruvate/malate (C), succinate (D), and palmitoylcarnitine (PC)/malate (E) as substrates. n = 5. In C, $p = 0.867$ (Baseline), $p = 0.742$ (Baseline), 0.766 (Pyruvate), 0.650 (ADP), and 0.903 (Oligomycin). In D, $p = 0.867$ (Baseline), 0.875 (Succinate), 0.609 (ADP), and 0.834 (Rotenone). In E, $p = 0.719$ (Baseline), 0.541 (PC), 0.527 (ADP) and 0.660 (Oligomycin).

The data are given as the mean ± SEM. Statistical analyses were carried out using two-sided t-test. One-asterisk indicates $p < 0.05$. Source data are provided as a Source Data file.

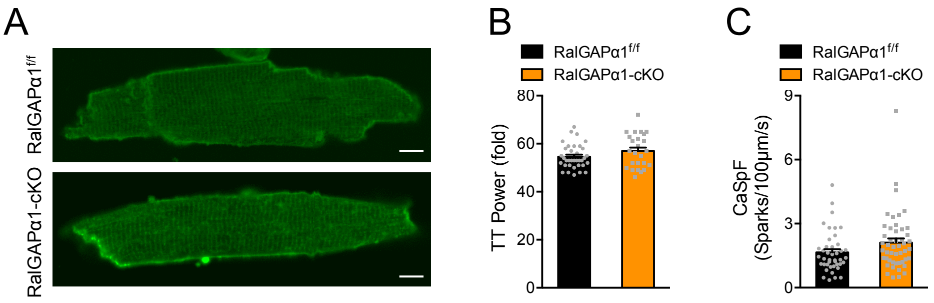

Supplementary Figure 4 Ca^{2+} sparks and t-tubule regularity in the RalGAPα1-cKO primary cardiomyocytes

A. Representative images for Di-8-ANEPPS staining in primary cardiomyocytes from the male RalGAPα1-cKO and RalGAPα1$^{f/f}$ mice (2-month-old). Bars indicate 5 μm in length.

B. Normalized TT power in primary cardiomyocytes isolated from the male RalGAPα1-cKO and RalGAPα1$^{f/f}$ mice (2-month-old). n = 25 (RalGAPα1-cKO) and 35 (RalGAPα1$^{f/f}$). $p = 0.124$.

C. Spontaneous calcium sparks in primary cardiomyocytes isolated from the 4-month-old male RalGAPα1-cKO (234 sparks from 48 cells) and RalGAPα1$^{f/f}$ mice (197 sparks from 42 cells). $p = 0.070$.

The data are given as the mean ± SEM. Statistical analyses were carried out using two-sided t-test. Source data are provided as a Source Data file.

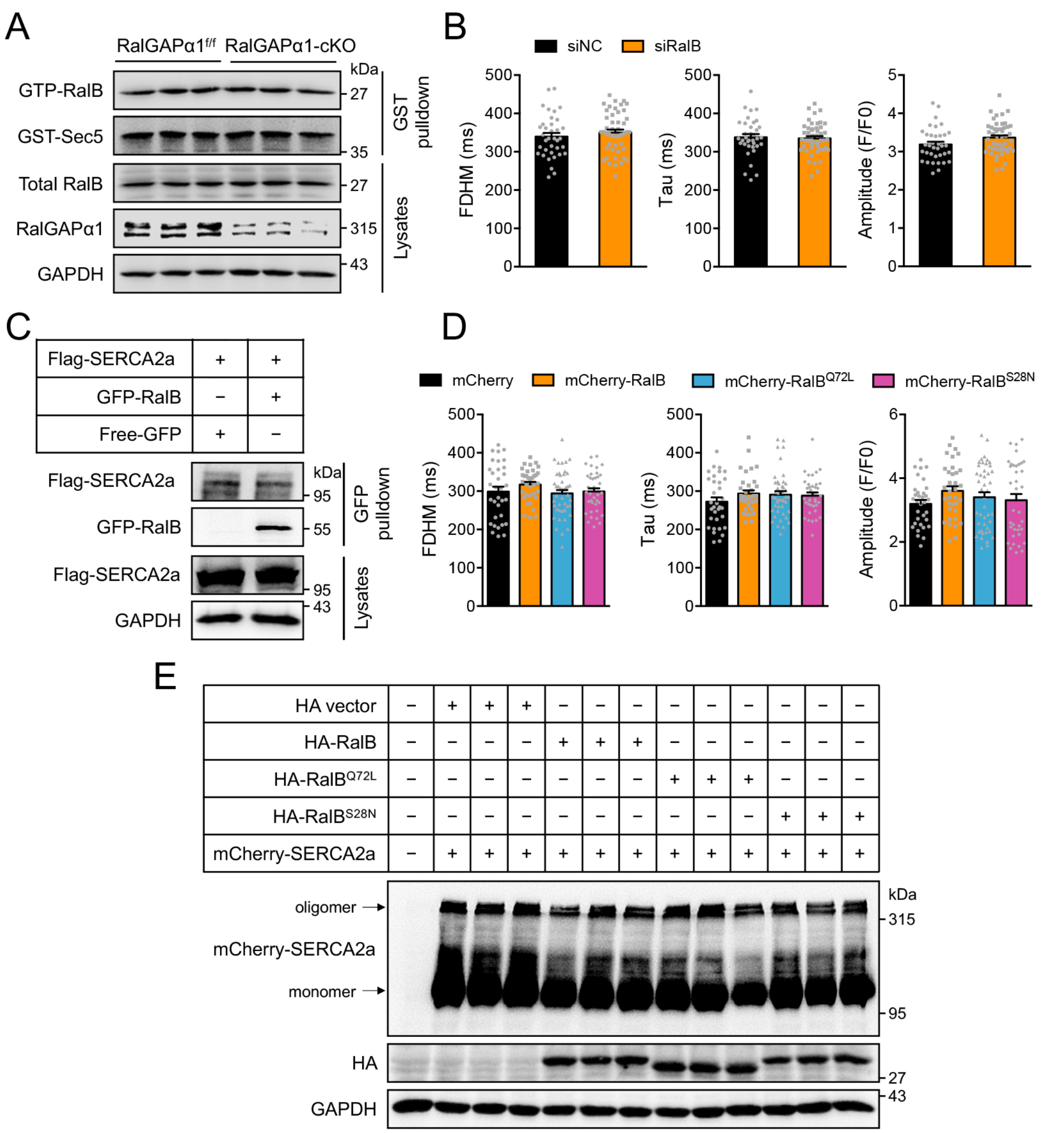

Supplementary Figure 5 Effects of RalB on Ca^{2+} homeostasis

A. GTP-bound RalB was measured in lysates of female RalGAPα1$^{f/f}$ and RalGAPα1-cKO hearts (2-month-old). RalB-GTP was pulled-down from heart lysates using GST-Sec5N as a bait, and detected via western blot using the anti-RalB antibody. Total RalB and RalGAPα1 were detected in heart lysates using GAPDH as a loading control.

B. Ca^{2+} transients in RalB-knockdown neonatal rat cardiomyocytes upon field stimulation. Amplitude, FDHM and Tau of Ca^{2+} transients were quantified from 36 (siNC) and 48 (siRalB) cells. $p = 0.391$ (FDHM), 0.773 (Tau), and 0.059 (Amplitude).

C. Flag-SERCA2a was expressed together with GFP-RalB or free GFP in HEK293 cells. After immunoprecipitation with the GFP antibody, Flag-SERCA2a was detected in the immunoprecipitates via western blot.

D. Ca^{2+} transients in neonatal rat cardiomyocytes expressing mCherry-RalB, mCherry-RalB^{Q72L}, mCherry-RalB^{S28N} or mCherry vector upon field stimulation. Amplitudes, FDHM and Tau of Ca^{2+} transients were quantified from 33 (vector), 35 (mCherry-RalB), 44 (mCherry-RalB^{Q72L}), and 37 (mCherry-RalB^{S28N}) cells. FDHM: $p = 0.984$ (mCherry-RalB^{S28N} vs mCherry), 0.189 (mCherry-RalB^{S28N} vs mCherry-RalB), and 0.701 (mCherry-RalB^{S28N} vs mCherry-RalB^{Q72L}). Tau: $p = 0.237$ (mCherry-RalB^{S28N} vs mCherry), 0.695 (mCherry-RalB^{S28N} vs mCherry-RalB), and 0.843 (mCherry-RalB^{S28N} vs mCherry-RalB^{Q72L}). Amplitude: $p = 0.617$ (mCherry-RalB^{S28N} vs mCherry), 0.195 (mCherry-RalB^{S28N} vs mCherry-RalB), and 0.656 (mCherry-RalB^{S28N} vs mCherry-RalB^{Q72L}).

E. Oligomerization of SERCA2a in HEK293 cells in which SERCA2a was co-expressed with HA-RalB, HA-RalB^{Q72L}, HA-RalB^{S28N} or an empty vector.

The data are given as the mean ± SEM. Statistical analyses were carried out using two-sided t-test for B and one-way ANOVA for D. Source data are provided as a Source Data file.

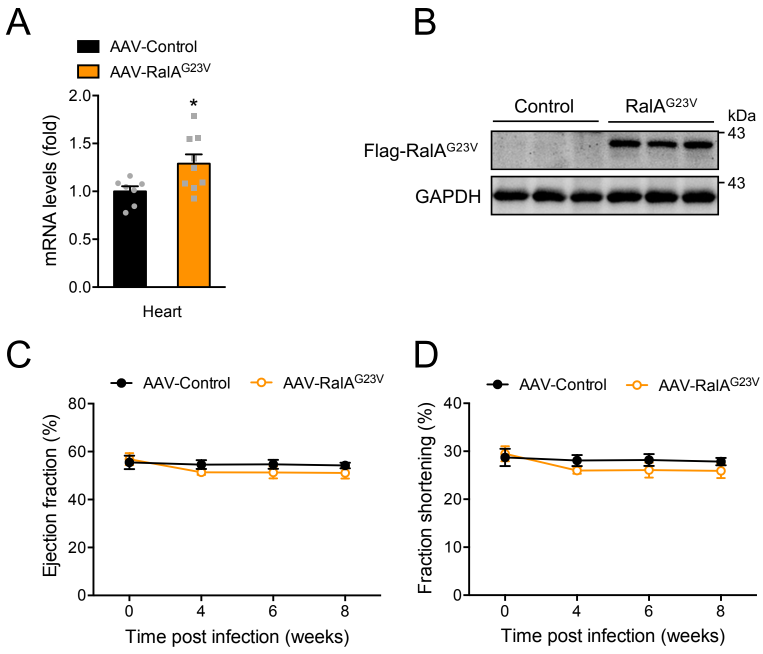

Supplementary Figure 6 AAV-mediated expression of Flag-RalA^{G23V} in mouse heart

A. *RalA* mRNA expression in the heart of AAV9-GFP or AAV9-RalA^{G23V} administered male C57BL/6J mice (4-month-old). n = 7 (AAV9-Control) and 9 (AAV9-RalA^{G23V}). $p = 0.029$.

B. Flag-RalA^{G23V} mutant protein expression in the heart of AAV9-GFP or AAV9-RalA^{G23V} administered male mice (4-month-old).

C-D. Cardiac function in AAV9-GFP or AAV9-RalA^{G23V} administered male mice. AAV9-GFP or AAV9-RalA^{G23V} was administered into 2-month-old male mice, and cardiac function was monitored for 2 months. C, ejection fraction. D, fraction shortening. n = 8 (AAV9-Control) and 7 (AAV9-RalA^{G23V}). Ejection fraction: p = 0.729 (0 W), 0.167 (4 W), 0.286 (6 W), and 0.246 (8 W). Fraction shortening: p = 0.758 (0 W), 0.152 (4 W), 0.300 (6 W), and 0.258 (8 W).

The data are given as the mean ± SEM. Statistical analyses were carried out using two-sided t-test. One-asterisk indicates $p < 0.05$. Source data are provided as a Source Data file.

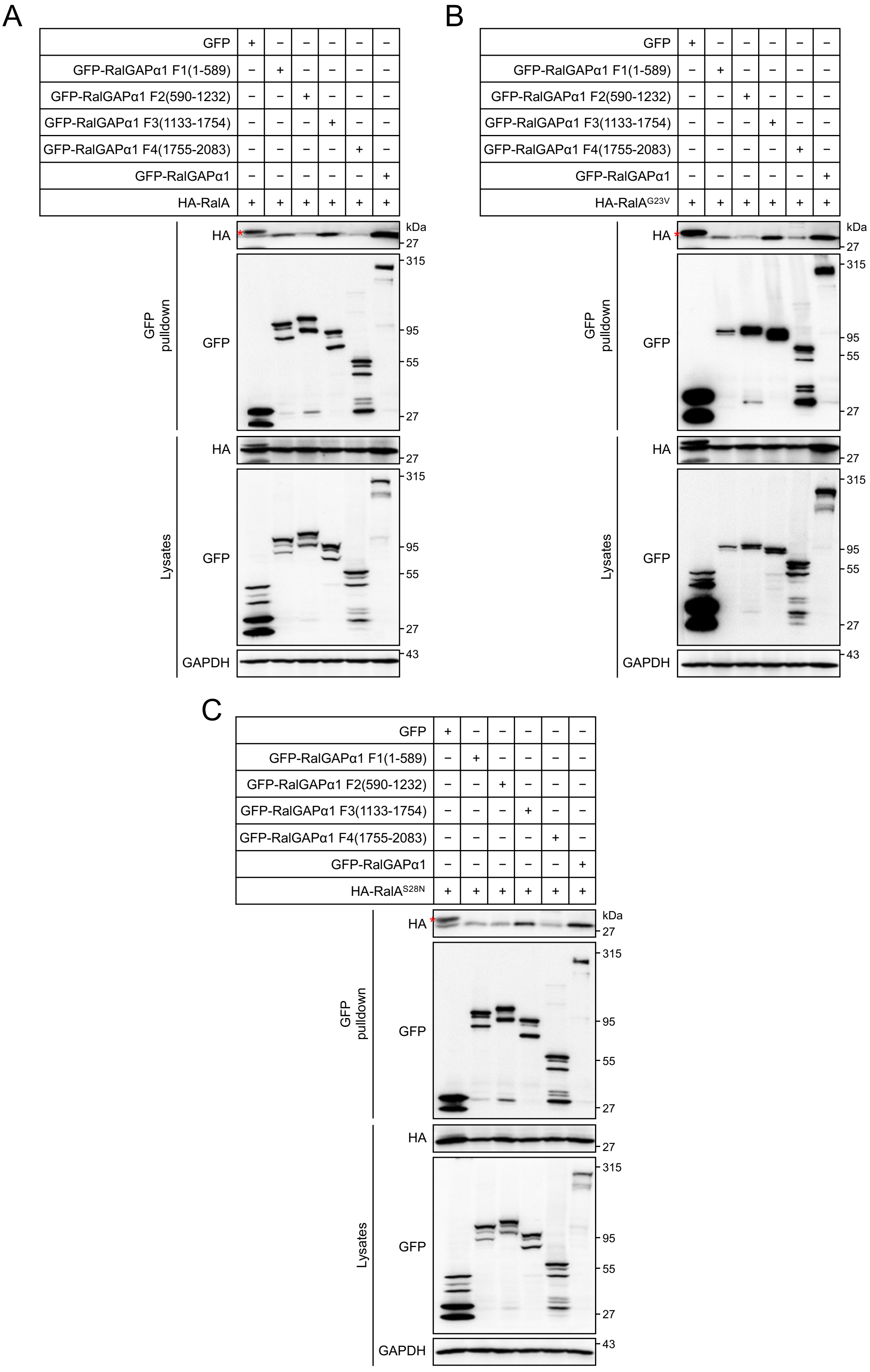

Supplementary Figure 7 RalA-interacting regions on RalGAPα1

A-C. Full-length and fragments of GFP-RalGAPα1 or GFP vector was co-expressed with HA-RalA (A), HA-RalA^{G23V} (B) or HA-RalA^{S28N} (C) in HEK293 cells. After immunoprecipitation with the GFP antibody, HA-RalA WT or mutants were detected in the immunoprecipitates via western blot. The red asterisk indicates a non-specific band (most likely due to cross-reaction of the HA antibody with a large quantity of free GFP) in the immunoprecipitates of free GFP. Source data are provided as a Source Data file.

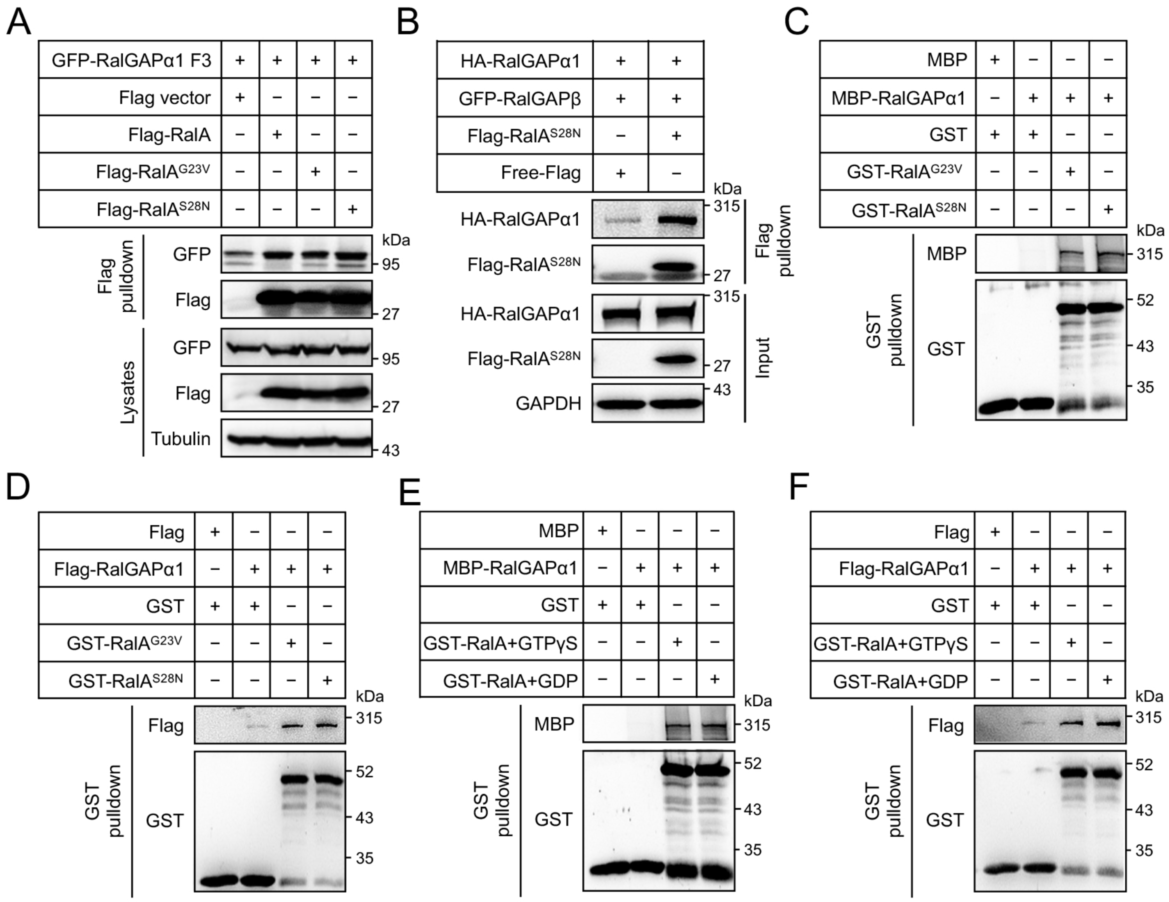

Supplementary Figure 8 Interaction of RalA and RalGAPα1

A. The GFP-RalGAPα1 F3 fragment spanning from 1133-1754aa was co-expressed with Flag-RalA, Flag-RalA^{G23V}, Flag-RalA^{S28N} or Flag vector in HEK293 cells. After immunoprecipitation with the Flag antibody, GFP-RalGAPα1 F3 was detected in the immunoprecipitates via western blot.

B. HA-RalGAPα1/GFP-RalGAPβ complex was co-expressed with Flag-RalA^{S28N} or free Flag in HEK293 cells. After immunoprecipitation with the Flag antibody, HA-RalGAPα1 was detected in the immunoprecipitates via western blot.

C. GST-RalA^{G23V} loaded with GTPγS, GST-RalA^{S28N} loaded with GDP and GST were used for *in vitro* pulldown of recombinant MBP-RalGAPα1. MBP-RalGAPα1 was expressed and purified from *E. coli*.

D. GST-RalA^{G23V} loaded with GTPγS, GST-RalA^{S28N} loaded with GDP and GST were used for *in vitro* pulldown of recombinant Flag-RalGAPα1. Flag-RalGAPα1 was expressed in HEK293 cells and purified using the Flag beads.

E. GST-RalA loaded with GTPγS or GDP was used for *in vitro* pulldown of recombinant MBP-RalGAPα1. MBP-RalGAPα1 was expressed and purified from *E. coli*.

F. GST-RalA loaded with GTPγS or GDP was used for *in vitro* pulldown of recombinant Flag-RalGAPα1. Flag-RalGAPα1 was expressed in HEK293 cells and purified using the Flag beads.

Source data are provided as a Source Data file.

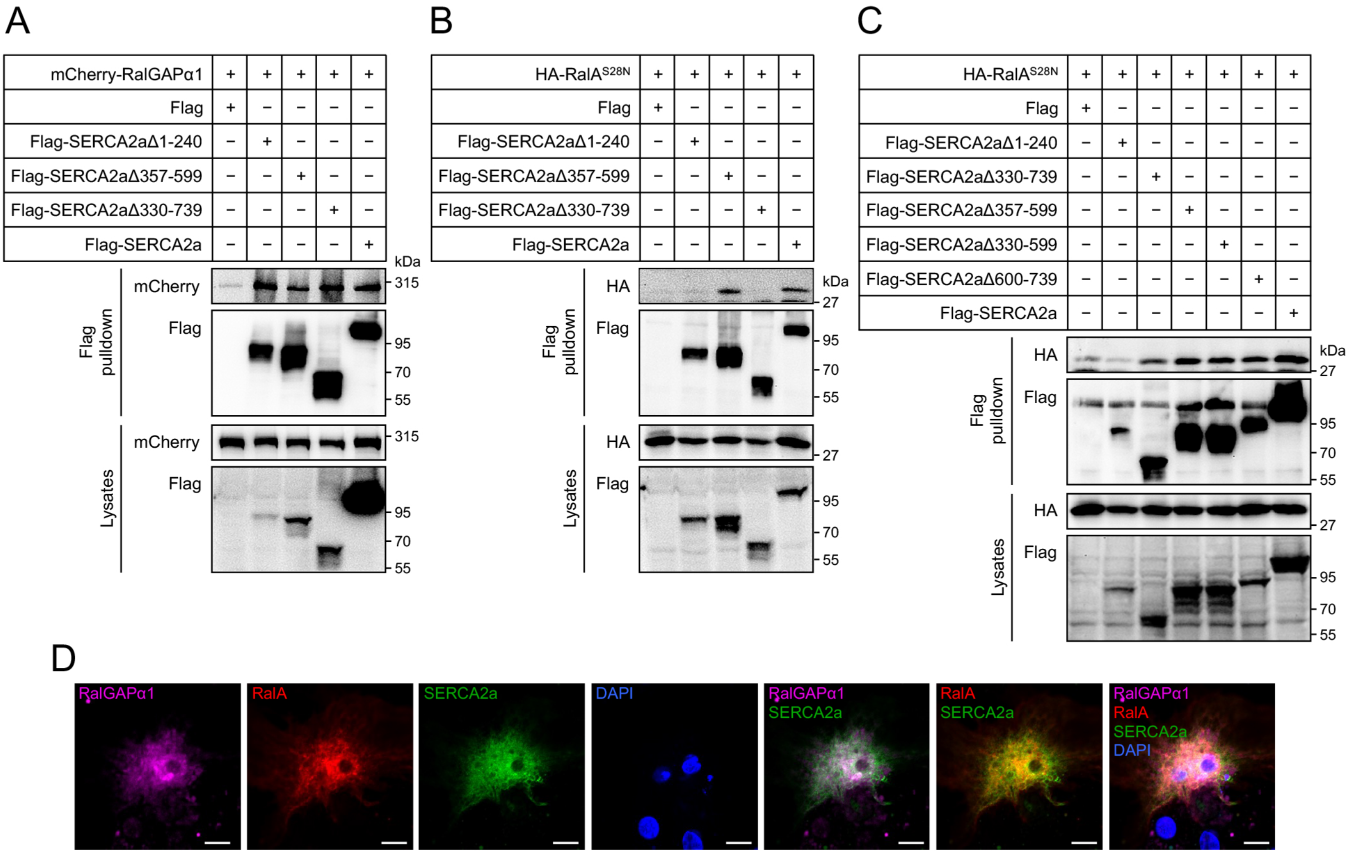

Supplementary Figure 9 Interaction and colocalization of RalGAPα1, RalA and SERCA2a

A. mCherry-RalGAPα1 was co-expressed with Flag-SERCA2a full-length or various deletion mutants in HEK293 cells. After immunoprecipitation with the Flag antibody, mCherry-RalGAPα1 was detected in the immunoprecipitates via western blot.

B-C. HA-RalA^{S28N} was co-expressed with Flag-SERCA2a full-length or various deletion mutants in HEK293 cells. After immunoprecipitation with the Flag antibody, HA-RalA^{S28N} was detected in the immunoprecipitates via western blot.

D. Colocalization of RalGAPα1, RalA and SERCA2a in cardiomyocytes. mCherry-RalA and GFP-SERCA2a were co-expressed in neonatal rat cardiomyocytes. Endogenous RalGAPα1 was stained with the specific antibody. Bars indicate 10 μm in length.

Source data are provided as a Source Data file.

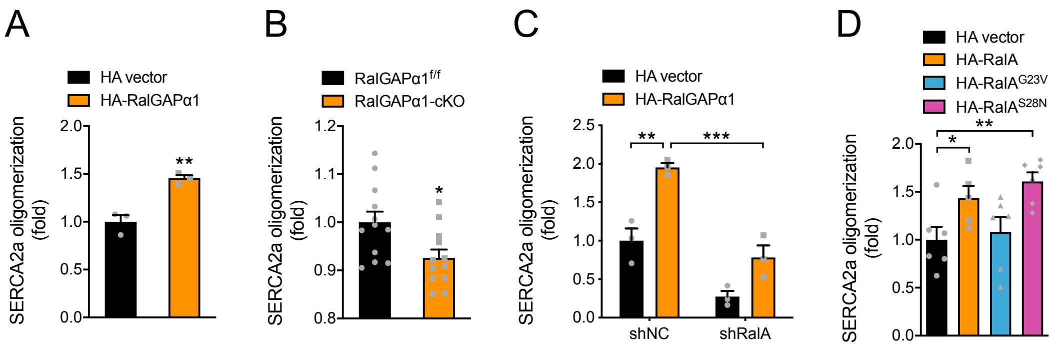

Supplementary Figure 10   Quantitation of SERCA2a oligomerization

Quantitation of oligomerization of SERCA2a in cells or tissues. Data were presented as fold changes of the ratio of oligomer SERCA2a to total SERCA2a.

A. Quantitation of oligomerization of SERCA2a in HEK293 cells in which SERCA2a was co-expressed with RalGAPα1 or an empty vector. Blots were shown in Fig. 5A. n = 3. $p = 0.0040$.

B, Quantitation of oligomerization of SERCA2a in the heart of female RalGAPα1-cKO mice and RalGAPα1$^{f/f}$ littermates (2-month-old). Representative blots were shown in Fig. 5B. n = 12. $p = 0.016$.

C. Quantitation of oligomerization of SERCA2a for immunoblots shown in Fig. 5D. n = 3. $p < 0.01$ (HA vector/shNC vs HA-RalGAPα1/shNC) and $p < 0.001$ (HA-RalGAPα1/shNC vs HA-RalGAPα1/shRalA).

D. Quantitation of oligomerization of SERCA2a in HEK293 cells in which SERCA2a was co-expressed with HA-RalA, HA-RalA^{G23V}, HA-RalA^{S28N} or an empty vector. Representative blots were shown in Fig. 5E. n = 6 (HA vector, HA-RalA^{G23V}, and HA-RalA^{S28N}) and 5 (HA-RalA). $p = 0.034$ (HA vector vs HA-RalA), 0.661 (HA vector vs HA-RalA^{G23V}) and 0.0033 (HA vector vs HA-RalA^{S28N}).

The data are given as the mean ± SEM. Statistical analyses were carried out using two-sided t-test for A and B, two-way ANOVA for C, and one-way ANOVA for D. One-asterisk indicates $p < 0.05$, two-asterisk indicates $p < 0.01$, and three-asterisk indicates $p < 0.001$. Source data are provided as a Source Data file.

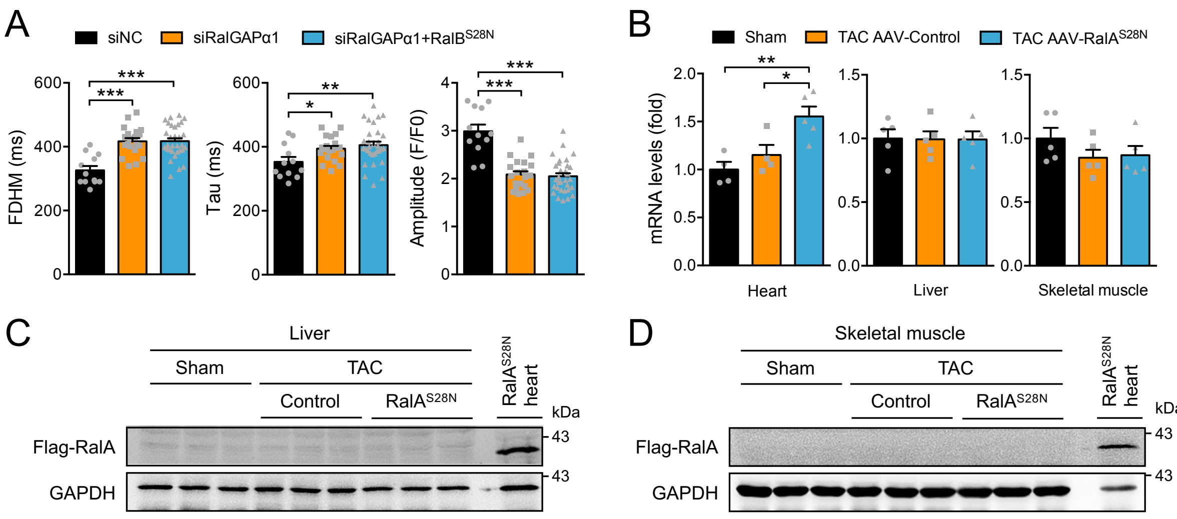

Supplementary Figure 11 AAV-mediated expression of Flag-RalA^{S28N} in mouse tissues

A. Ca^{2+} transients upon field stimulation in RalGAPα1-depleted neonatal rat cardiomyocytes that were transfected with or without RalB^{S28N}. Amplitude, FDHM and Tau of Ca^{2+} transients were quantified from 12 (siNC), 20 (siRalGAPα1) and 31 (siRalGAPα1+RalB^{S28N}) cells. FDHM: $p < 0.0001$ (siNC vs siRalGAPα1, siNC vs siRalGAPα1+RalB^{S28N}), and $p = 0.977$ (siRalGAPα1 vs siRalGAPα1+RalB^{S28N}). Tau: $p = 0.034$ (siNC vs siRalGAPα1), 0.0044 (siNC vs siRalGAPα1+RalB^{S28N}) and 0.459 (siRalGAPα1 vs siRalGAPα1+RalB^{S28N}). Amplitude: $p < 0.0001$ (siNC vs siRalGAPα1, siNC vs siRalGAPα1+RalB^{S28N}), and $p = 0.750$ (siRalGAPα1 vs siRalGAPα1+RalB^{S28N}).

B. *RalA* mRNA expression in the heart, liver and skeletal muscle of AAV9-GFP or AAV9-RalA^{S28N} administered male mice (20-week-old). n = 4 (Sham and TAC AAV9-Control) and 5 (TAC AAV9-RalA^{S28N}). Heart: $p = 0.323$ (Sham vs TAC AAV9-Control), 0.0025 (Sham vs TAC AAV9-RalA^{S28N}) and 0.016 (TAC AAV9-Control vs TAC AAV9-RalA^{S28N}). Liver: $p = 0.945$ (Sham vs TAC AAV9-Control), 0.940 (Sham vs TAC AAV9-RalA^{S28N}) and 0.995 (TAC AAV9-Control vs TAC AAV9-RalA^{S28N}). Skeletal muscle: $p = 0.173$ (Sham vs TAC AAV9-Control), 0.232 (Sham vs TAC AAV9-RalA^{S28N}) and 0.853 (TAC AAV9-Control vs TAC AAV9-RalA^{S28N}).

C-D. Flag-RalA^{S28N} mutant protein expression in the liver (C) and skeletal muscle (D) of AAV9-GFP or AAV9-RalA^{S28N} administered male mice (20-week-old).

The data are given as the mean ± SEM. Statistical analyses were carried out using one-way ANOVA. One-asterisk indicates $p < 0.05$, two-asterisk indicates $p < 0.01$, and three-asterisk indicates $p < 0.001$. Source data are provided as a Source Data file.

Supplementary figure 1A
（Uncropped blots）

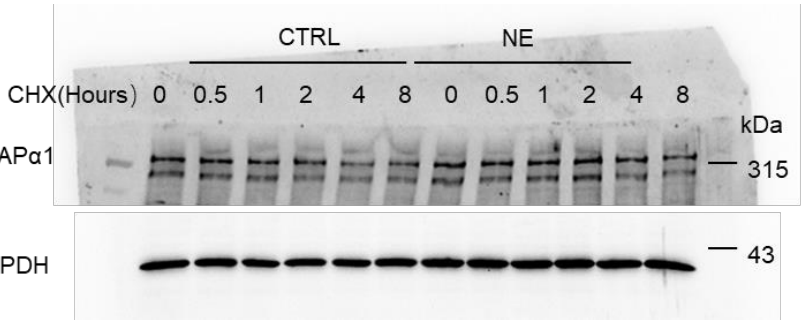

Supplementary figure 1C
（Uncropped blots）

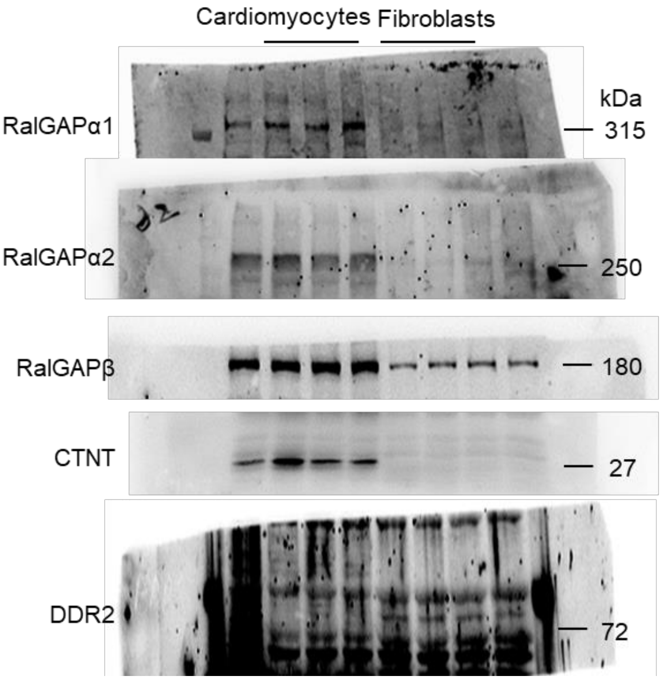

Supplementary figure 2B
(Uncropped blots)

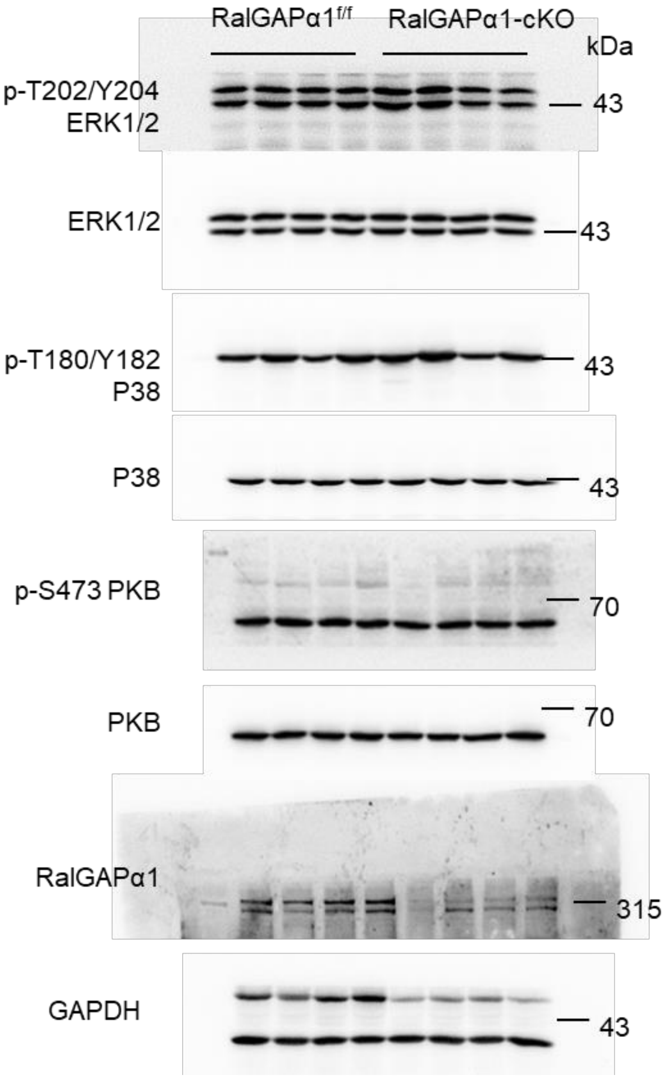

Supplementary figure 2D
（Uncropped blots）

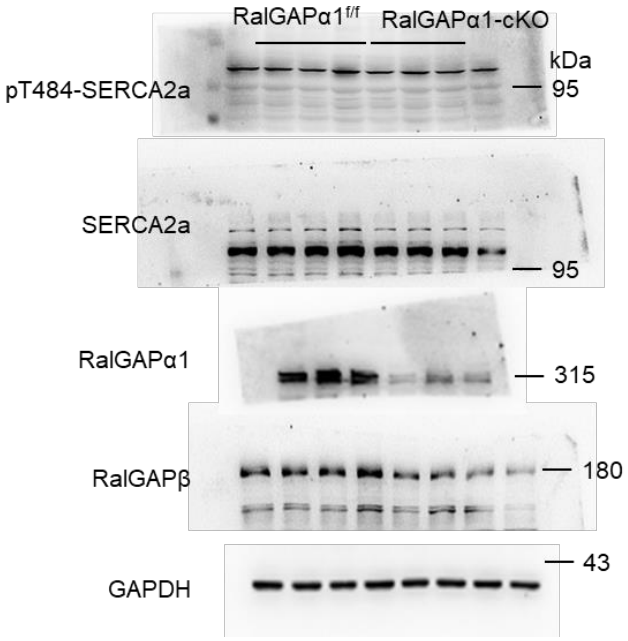

Supplementary figure 5A
(Uncropped blots)

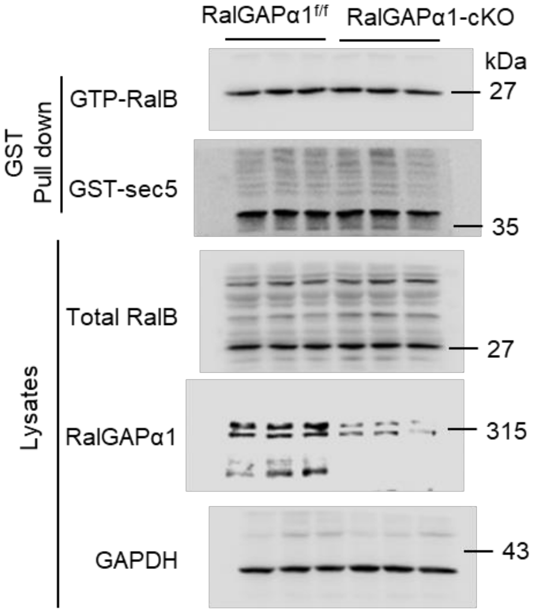

Supplementary figure 5C
(Uncropped blots)

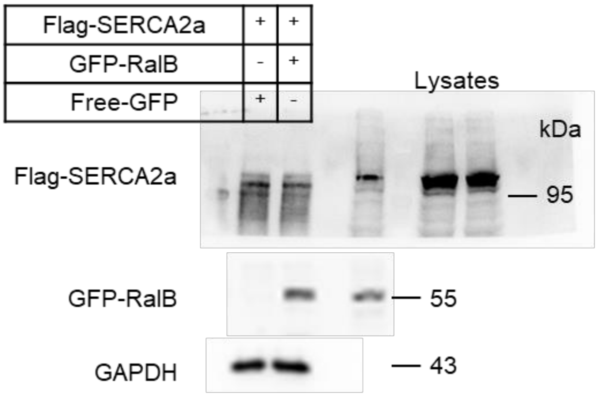

Supplementary figure 5E
（Uncropped blots）

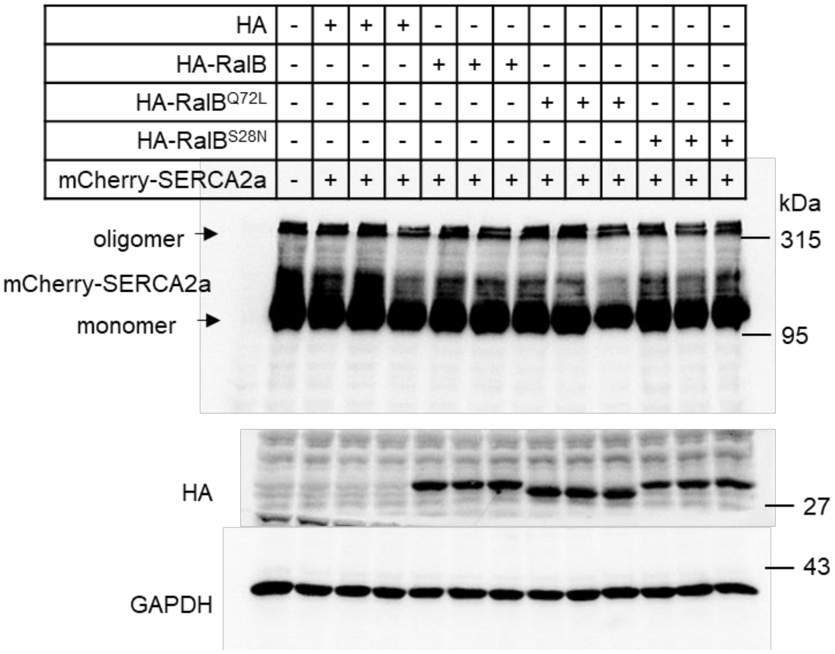

Supplementary figure 6B
（Uncropped blots）

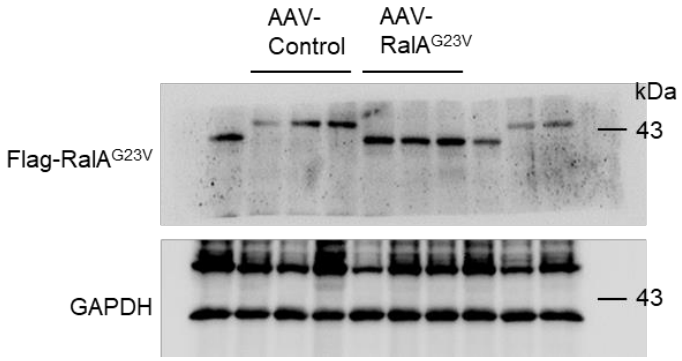

Supplementary figure 7A
（Uncropped blots）

GFP	+	-	-	-	-	-
GFP-RalGAPα1 F1(1-589)	-	+	-	-	-	-
GFP-RalGAPα1 F2(590-1232)	-	-	+	-	-	-
GFP-RalGAPα1 F3(1133-1754)	-	-	-	+	-	-
GFP-RalGAPα1 F4(1755-2083)	-	-	-	-	+	-
GFP-RalGAPα1	-	-	-	-	-	+
HA-RalA	+	+	+	+	+	+

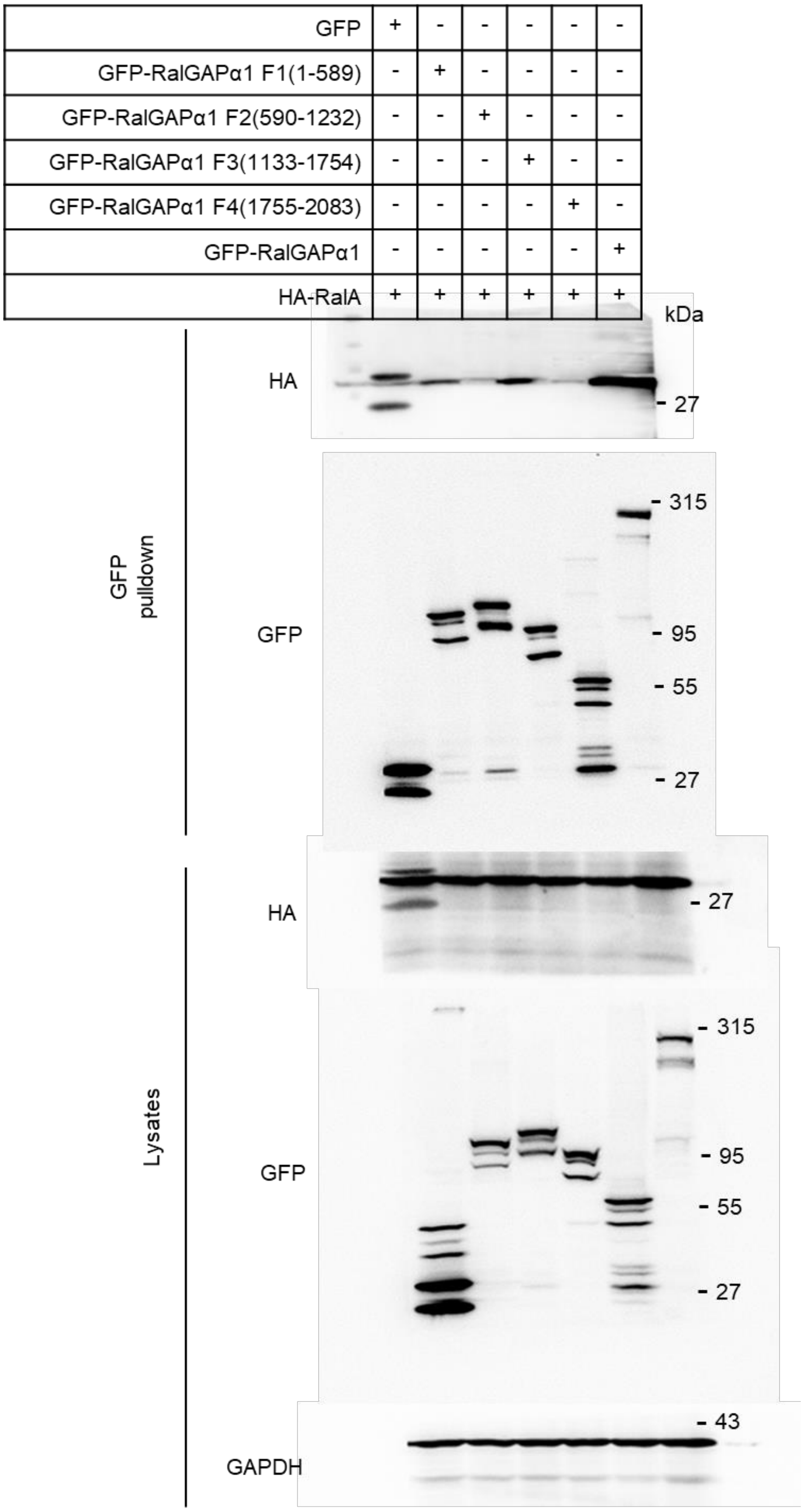

Supplementary figure 7B
（Uncropped blots）

GFP	+	-	-	-	-	-
GFP-RalGAPα1 F1(1-589)	-	+	-	-	-	-
GFP-RalGAPα1 F2(590-1232)	-	-	+	-	-	-
GFP-RalGAPα1 F3(1133-1754)	-	-	-	+	-	-
GFP-RalGAPα1 F4(1755-2083)	-	-	-	-	+	-
GFP-RalGAPα1	-	-	-	-	-	+
HA-RalA^{G23V}	+	+	+	+	+	+

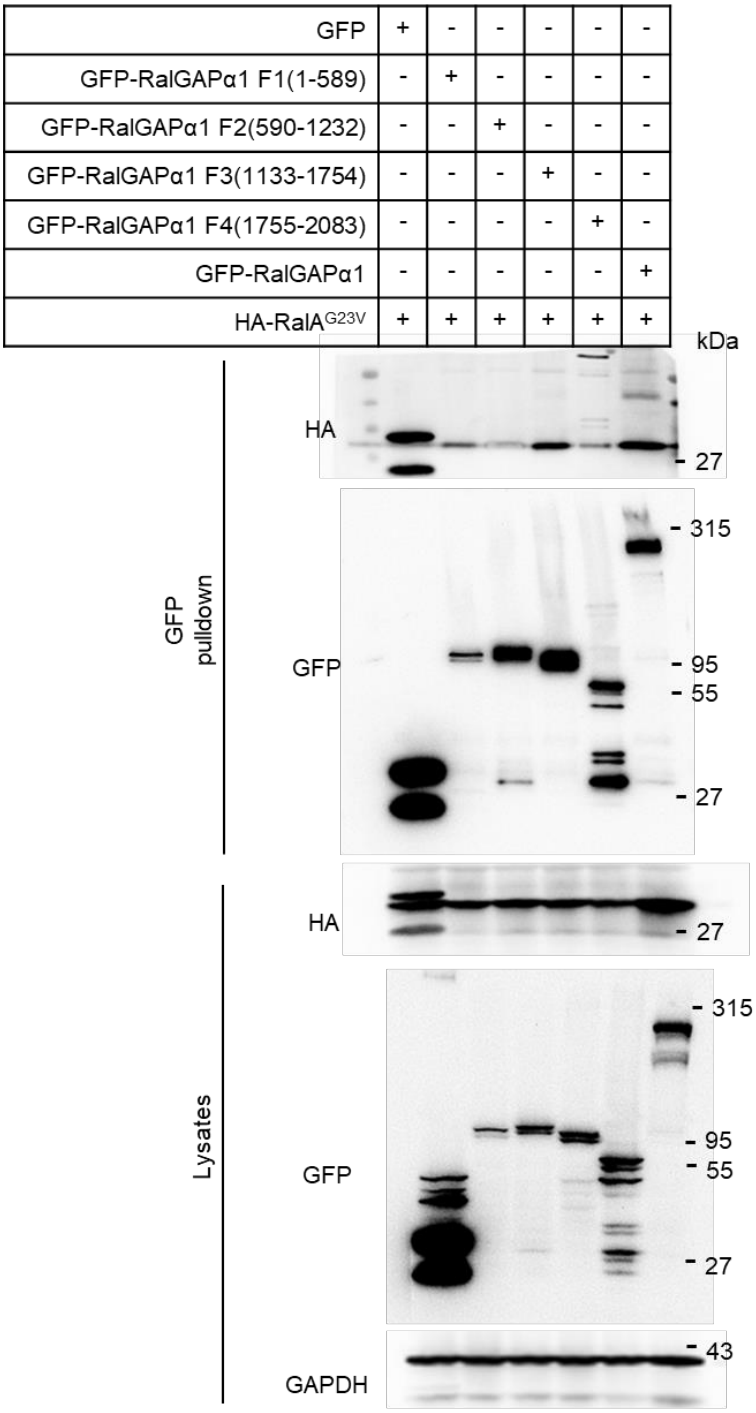

Supplementary figure 7C
（Uncropped blots）

GFP	+	-	-	-	-	-
GFP-RalGAPα1 F1(1-589)	-	+	-	-	-	-
GFP-RalGAPα1 F2(590-1232)	-	-	+	-	-	-
GFP-RalGAPα1 F3(1133-1754)	-	-	-	+	-	-
GFP-RalGAPα1 F4(1755-2083)	-	-	-	-	+	-
GFP-RalGAPα1	-	-	-	-	-	+
HA-RalA^{S28N}	+	+	+	+	+	+

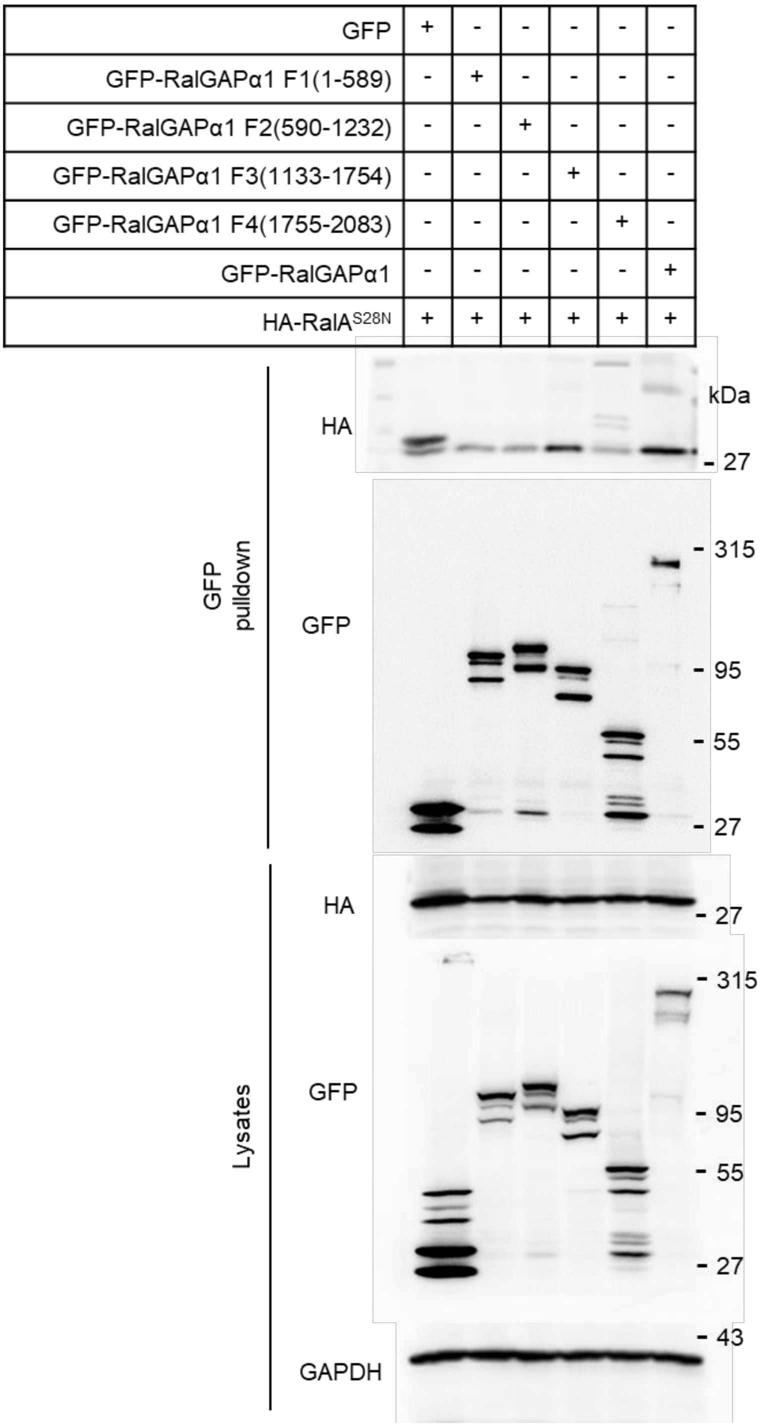

Supplementary figure 8A
（Uncropped blots）

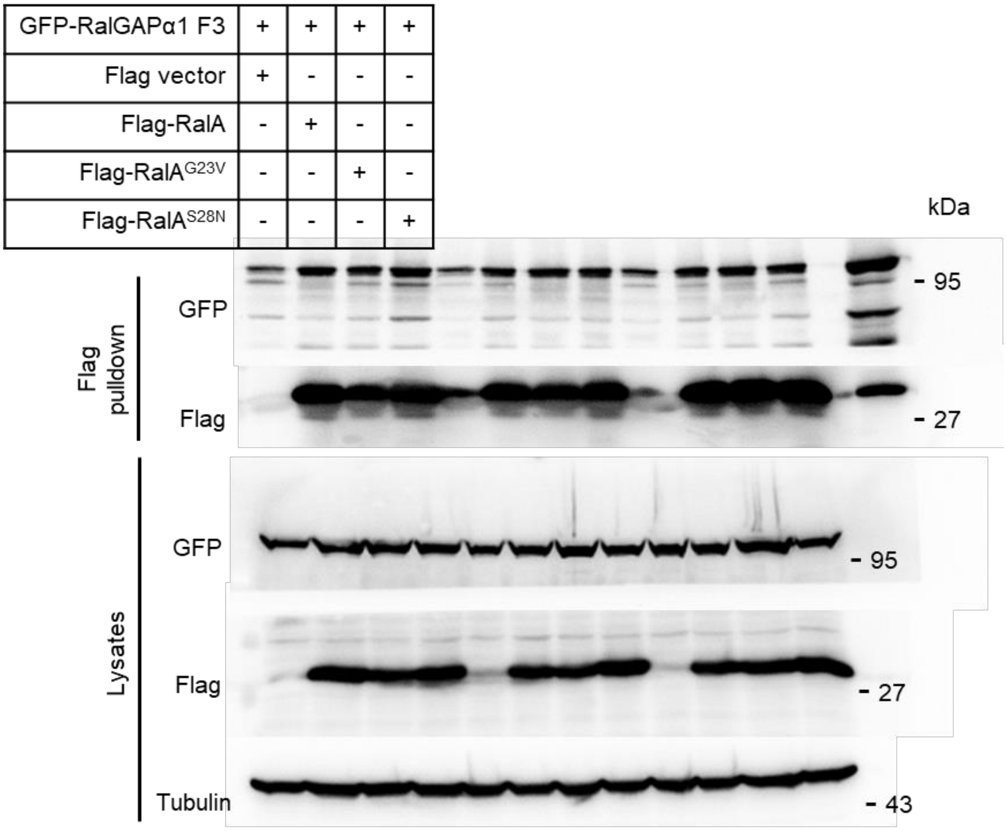

Supplementary figure 8B
（Uncropped blots）

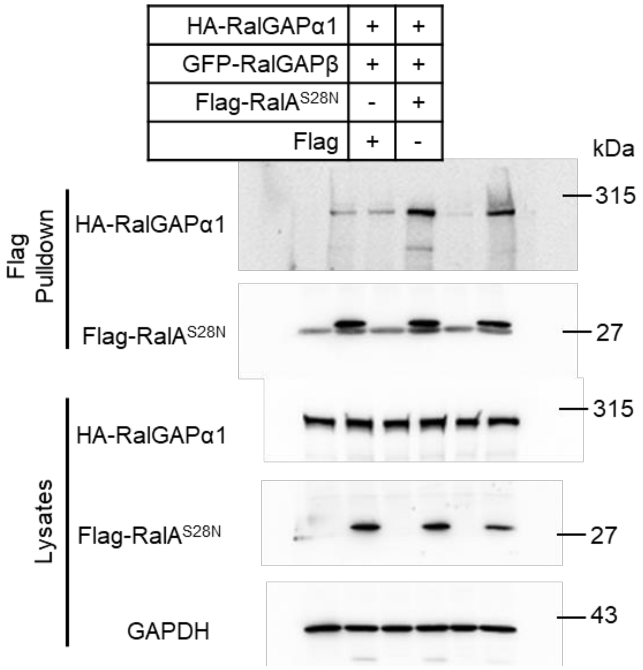

Supplementary figure 8C （Uncropped blots）

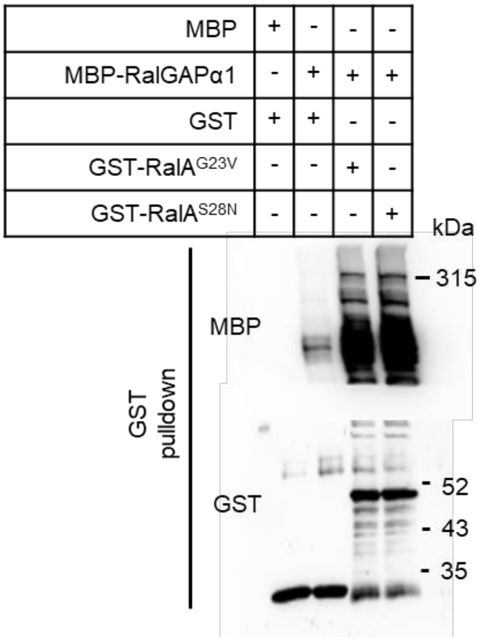

Supplementary figure 8D （Uncropped blots）

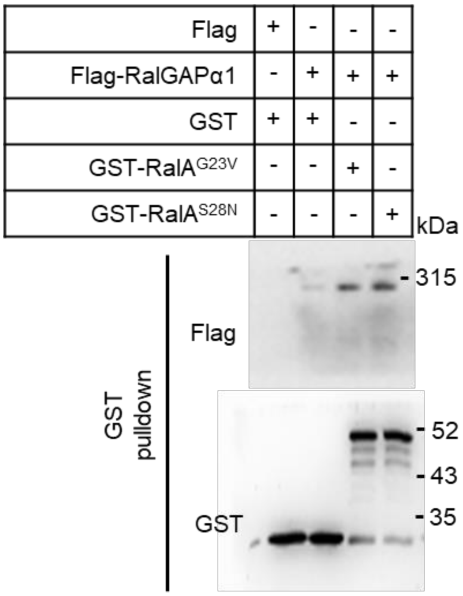

Supplementary figure 8E （Uncropped blots）

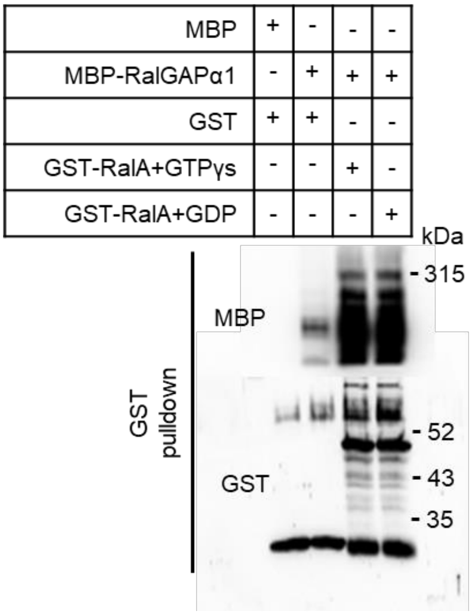

Supplementary figure 8F （Uncropped blots）

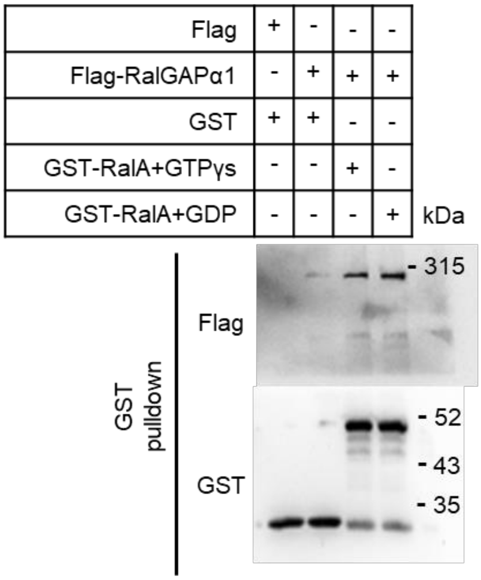

Supplementary figure 9A
（Uncropped blots）

mCherry-RalGAPα1	+	+	+	+	+
Flag	+	-	-	-	-
Flag-Serca2aΔ1-240	-	+	-	-	-
Flag-Serca2aΔ357-599	-	-	+	-	-
Flag-Serca2aΔ330-739	-	-	-	+	-
Flag-Serca2a	-	-	-	-	+

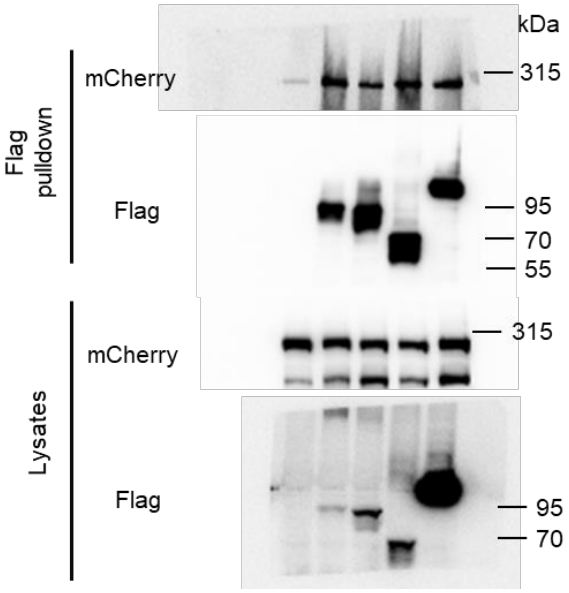

Supplementary figure 9B
（Uncropped blots）

HA-RalA^{S28N}	+	+	+	+	+
Flag	+	-	-	-	-
Flag-Serca2aΔ1-240	-	+	-	-	-
Flag-Serca2aΔ357-599	-	-	+	-	-
Flag-Serca2aΔ330-739	-	-	-	+	-
Flag-Serca2a	-	-	-	-	+

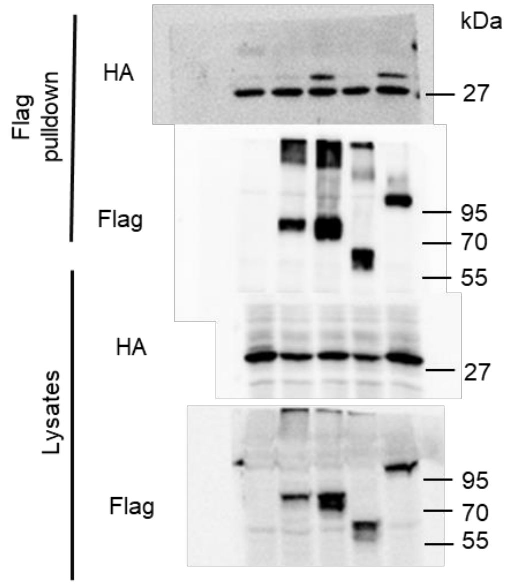

Supplementary figure 9C
（Uncropped blots）

HA-RalA^{S28N}	+	+	+	+	+	+	+
Flag	+	-	-	-	-	-	-
Flag-Serca2aΔ1-240	-	+	-	-	-	-	-
Flag-Serca2aΔ330-739	-	-	+	-	-	-	-
Flag-Serca2aΔ357-599	-	-	-	+	-	-	-
Flag-Serca2aΔ330-599	-	-	-	-	+	-	-
Flag-Serca2aΔ600-739	-	-	-	-	-	+	-
Flag-Serca2a	-	-	-	-	-	-	+

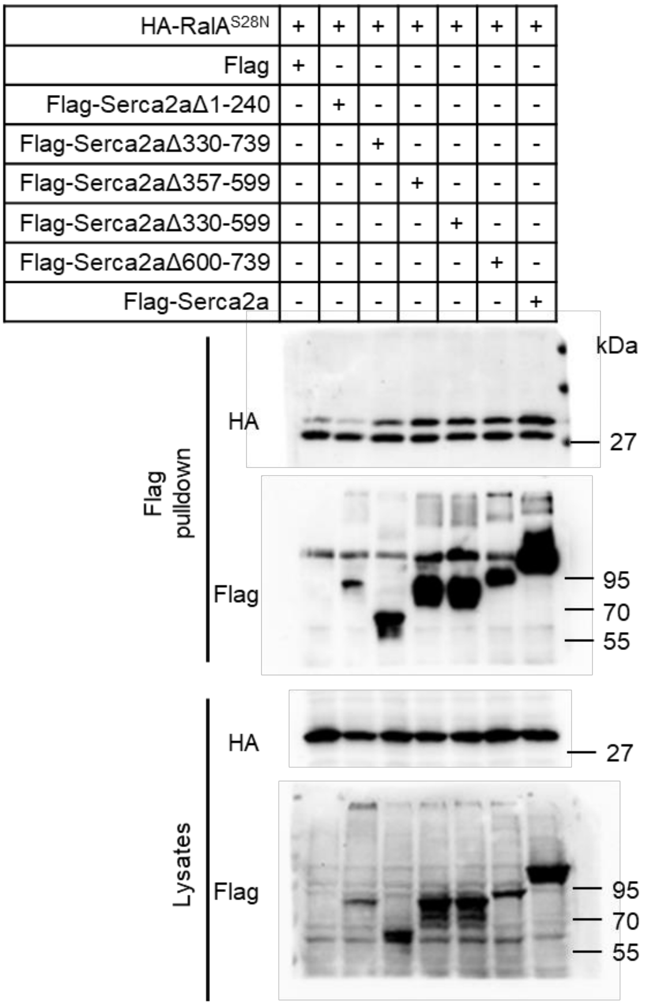

Supplementary figure 11C
（Uncropped blots）

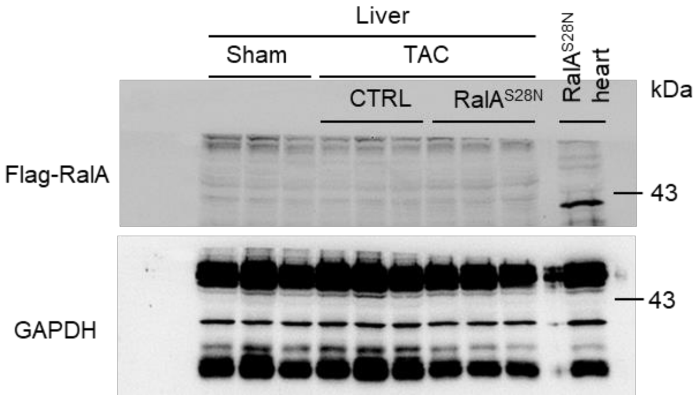

Supplementary figure 11D
（Uncropped blots）

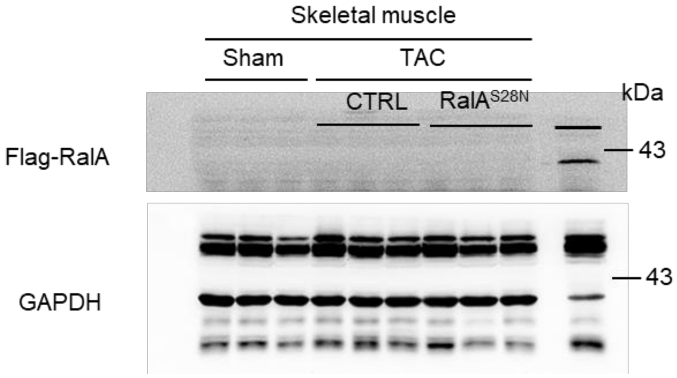

Supplement: Supplementary file 1 — Updated Supplementary file [file 41467_2022_31992_MOESM1_ESM.pdf]
