# Supplementary material for: The RalGAPα1–RalA signal module protects cardiac function through regulating calcium homeostasis
Source: Nat Commun. 2022 Jul 25;13:4278. doi: 10.1038/s41467-022-31992-z (PMC9314365; doi:10.1038/s41467-022-31992-z)
Supplement: Supplementary file 2 — Description of additional supplementary items [file 41467_2022_31992_MOESM2_ESM.docx]

**Supplementary Data 1**

**Proteins co-immunoprecipitated with GFP-RalGAPα1**. GFP-RalGAPα1 fusion protein was expressed in HEK293 cells together with HA-RalGAPβ. Cells transfected with empty vectors were used as a control. Immunoprecipitation was performed using the GFP-binder, and resultant immunoprecipitates were subjected to separation via SDS-PAGE. The protein bands in the GFP-RalGAPα1 immunoprecipitates were excised, subjected to trypsin-digestion, and identified via mass-spectrometry. Protein band 2 was identified as GFP-RalGAPα1, and band 3 was identified as HA-RalGAPβ. Proteins in Bands 1, 4-8 were listed in this table.
