# Supplementary material for: The RalGAPα1–RalA signal module protects cardiac function through regulating calcium homeostasis
Source: Nat Commun. 2022 Jul 25;13:4278. doi: 10.1038/s41467-022-31992-z (PMC9314365; doi:10.1038/s41467-022-31992-z)
Supplement: Supplementary file 4 — Reporting Summary [file 41467_2022_31992_MOESM4_ESM.pdf]

nature portfolio

Corresponding author(s): Shuai Chen

Last updated by author(s): Jun 5, 2022

Reporting Summary

Nature Portfolio wishes to improve the reproducibility of the work that we publish. This form provides structure for consistency and transparency in reporting. For further information on Nature Portfolio policies, see our Editorial Policies and the Editorial Policy Checklist.

Statistics

For all statistical analyses, confirm that the following items are present in the figure legend, table legend, main text, or Methods section.

n/a	Confirmed	
☐	☒	The exact sample size (n) for each experimental group/condition, given as a discrete number and unit of measurement
☐	☒	A statement on whether measurements were taken from distinct samples or whether the same sample was measured repeatedly
☐	☒	The statistical test(s) used AND whether they are one- or two-sided<br>*Only common tests should be described solely by name; describe more complex techniques in the Methods section.*
☒	☐	A description of all covariates tested
☒	☐	A description of any assumptions or corrections, such as tests of normality and adjustment for multiple comparisons
☐	☒	A full description of the statistical parameters including central tendency (e.g. means) or other basic estimates (e.g. regression coefficient) AND variation (e.g. standard deviation) or associated estimates of uncertainty (e.g. confidence intervals)
☐	☒	For null hypothesis testing, the test statistic (e.g. F, t, r) with confidence intervals, effect sizes, degrees of freedom and P value noted<br>*Give P values as exact values whenever suitable.*
☒	☐	For Bayesian analysis, information on the choice of priors and Markov chain Monte Carlo settings
☒	☐	For hierarchical and complex designs, identification of the appropriate level for tests and full reporting of outcomes
☒	☐	Estimates of effect sizes (e.g. Cohen's d, Pearson's r), indicating how they were calculated

Our web collection on statistics for biologists contains articles on many of the points above.

Software and code

Policy information about availability of computer code

Data collection	No software was used to collect the data.
Data analysis	All commercial and open source code used to analyse the data are described, including ImageJ software (version 1.46) with a plugin TTorg (http://mirror.imagej.net/plugins/ttorg), IDL 5.5 (Harris Geospatial Solutions), Clampfit 10.4 (Molecular Devices), ProteinPilot Software (version 4.5, AB Sciex) and Prism software (Version 9.0 GraphPad).

For manuscripts utilizing custom algorithms or software that are central to the research but not yet described in published literature, software must be made available to editors and reviewers. We strongly encourage code deposition in a community repository (e.g. GitHub). See the Nature Portfolio guidelines for submitting code & software for further information.

Data

Policy information about availability of data

All manuscripts must include a data availability statement. This statement should provide the following information, where applicable:

- Accession codes, unique identifiers, or web links for publicly available datasets
- A description of any restrictions on data availability
- For clinical datasets or third party data, please ensure that the statement adheres to our policy

All data generated or analysed during this study are included in this published article and its supplementary information files. Proteomics data have been deposited in the MassIVE Repository in University of California, San Diego (ftp://massive.ucsd.edu/MSV000089597).

Field-specific reporting

Please select the one below that is the best fit for your research. If you are not sure, read the appropriate sections before making your selection.

- [x] Life sciences
- [ ] Behavioural & social sciences
- [ ] Ecological, evolutionary & environmental sciences

For a reference copy of the document with all sections, see nature.com/documents/nr-reporting-summary-flat.pdf

Life sciences study design

All studies must disclose on these points even when the disclosure is negative.

Sample size	No statistical methods were used to predetermine sample size due to the nature of this study. The sample size follows common standards employing three or more biological replicates, which is based on extensive laboratory experience and literature in the field. Sample size is reported in the legends for all figures.
Data exclusions	No data were excluded from the analyses.
Replication	Results shown in the manuscript are representative of at least two similar experiments. All the attempts at replication were successful.
Randomization	Samples were randomly allocated into experimental groups.
Blinding	The investigators were blinded to group allocation during data collection and analysis.

Reporting for specific materials, systems and methods

We require information from authors about some types of materials, experimental systems and methods used in many studies. Here, indicate whether each material, system or method listed is relevant to your study. If you are not sure if a list item applies to your research, read the appropriate section before selecting a response.

Materials & experimental systems

n/a	Involved in the study	
☐	☒	Antibodies
☐	☒	Eukaryotic cell lines
☒	☐	Palaeontology and archaeology
☐	☒	Animals and other organisms
☒	☐	Human research participants
☒	☐	Clinical data
☒	☐	Dual use research of concern

Methods

n/a	Involved in the study	
☒	☐	ChIP-seq
☒	☐	Flow cytometry
☒	☐	MRI-based neuroimaging

Antibodies

Antibodies used

anti-RalA （Cell Signaling Technology, Cat: #4799, dilution: 1:1000）
anti-RalB (Abclonal, Cat: WH079944, dilution: 1:1000)
anti-β-MHC (Sigma, Cat: t9283, dilution: 1:1000)
anti-cTnT (Thermo Fisher, Cat: MA5-12960, dilution: 1:1000)
anti-DDR2 (Santa Cruz Biotechnology, Cat: sc-8989, dilution: 1:1000)
anti-SERCA2a (Proteintech, Cat: 13985-1-AP, dilution: 1:1000)
anti-pT202/Y204-ERK1/2 (Cell Signaling Technology, Cat: 4370, dilution: 1:1000)
anti-ERK1/2 (Cell Signaling Technology, Cat: 4695, dilution: 1:1000)
anti-pT180/Y182-p38 (Cell Signaling Technology, Cat: 4511, dilution: 1:1000)
anti-p38 (Cell Signaling Technology, Cat: 8690, dilution: 1:1000)
anti-pS473-PKB (Cell Signaling Technology, Cat: 9271S, dilution: 1:1000)
anti-PKB (Cell Signaling Technology, Cat: 9272S, dilution: 1:1000)
anti-RalGAPα1 (Invitrogen, Cat: PA5-51467, dilution: 1:200 for immunofluorescence study)
anti-PDI (Cell Signaling Technology, Cat: 3501S, dilution: 1:1000)
anti-mCherry (Proteintech Cat: 26765-1-AP, dilution: 1:1000)
anti-HA (Cell Signaling Technology, Cat: #3724, dilution: 1:1000)
anti-Flag (Sigma, Cat: F9291, dilution: 1:1000)
anti-GFP (Santa Cruz Biotechnology, Cat: sc-9996, dilution: 1:1000)
anti-GST (Abclonal, Cat: AE001, dilution: 1:1000)
anti-GAPDH (Proteintech, Cat: 60004-1, dilution: 1:1000)
anti-RalGAPα1 (provided by Dr. Hisanori Horiuchi (Tohoku University, Japan), dilution: 1:1000)

	anti-RalGAPα2 (provided by Dr. Hisanori Horiuchi (Tohoku University, Japan), dilution: 1:1000)<br>anti-RalGAPβ (provided by Dr. Hisanori Horiuchi (Tohoku University, Japan), dilution: 1:1000)<br>anti-pThr484-SERCA2a (homemade, previously reported by Quan et al (Circulation Research, 2019, 124(5): 712-726), dilution: 1μg/ml)<br>Goat anti-Rabbit IgG, horseradish peroxidase (HRP) conjugated secondary antibody (Jackson ImmunoResearch Labs, Cat No. 111-035-003, dilution: 1:5000)<br>Mouse anti-Rabbit IgG, horseradish peroxidase (HRP) conjugated secondary antibody (Jackson ImmunoResearch Labs, Cat No. 211-002-171, dilution: 1:5000)<br>Goat anti-Mouse IgG, horseradish peroxidase (HRP) conjugated secondary antibody (Jackson ImmunoResearch Labs, Cat No. 115-035-003, dilution: 1:5000)<br>Goat anti-Mouse IgG, horseradish peroxidase (HRP) conjugated secondary antibody (Jackson ImmunoResearch Labs, Cat No. 115-005-174, dilution: 1:5000)
Validation	Relevant data for validation of all primary antibodies for immunoblotting or immunofluorescence in this study are presented on the manufactures' websites. The PAS Sepharose beads and GFP-Trap®-agarose were used for immunoprecipitation, with validation data shown on the manufactures' websites.The RalGAPα1, RalGAPα2 and RalGAPβ antibodies were previously validated by Ryutaro Shirakawa et al (J Biol Chem, 2009, 284(32): 21580-8). The pThr484-SERCA2a was previously validated by Quan et al (Circulation Research, 2019, 124(5): 712-726).

Eukaryotic cell lines

Policy information about cell lines

Cell line source(s)	Human embryonic kidney HEK293 cell was obtained from the Cell Resource Center, Chinese Academy of Medical Sciences and Peking Union Medical College (China).
Authentication	Cell lines were not authenticated using STR profiling, Karyotyping, DNA barcoding, PCR assays with species-specific primers, etc technics. Cell lines were authenticated in our lab via routine observation of cell morphology under the microscope.
Mycoplasma contamination	All cell lines were tested negative for mycoplasma contamination.
Commonly misidentified lines (See ICLAC register)	No commonly misidentified cell lines were used in the study.

Animals and other organisms

Policy information about studies involving animals; ARRIVE guidelines recommended for reporting animal research

Laboratory animals	Mice (strain C57Bl/6J, both male and female) with age from 1.5 to 9 months and neonatal rats (Sprague Dawley, both male and female) were used in this study.
Wild animals	The study did not involve wild animals.
Field-collected samples	The study did not involve samples collected from the field.
Ethics oversight	The Institutional Animal Care and Use Committee (IACUC) at Model Animal Research Center of Nanjing University approved all animal procedures involving mice and rats in this study.

Note that full information on the approval of the study protocol must also be provided in the manuscript.
